# NudC L279P Mutation Destabilizes Filamin A by Inhibiting the Hsp90 Chaperoning Pathway and Suppresses Cell Migration

**DOI:** 10.3389/fcell.2021.671233

**Published:** 2021-06-18

**Authors:** Min Liu, Zhangqi Xu, Cheng Zhang, Chunxia Yang, Jiaxing Feng, Yiqing Lu, Wen Zhang, Wenwen Chen, Xiaoyang Xu, Xiaoxia Sun, Mingyang Yang, Wei Liu, Tianhua Zhou, Yuehong Yang

**Affiliations:** ^1^Department of Cell Biology, and Institute of Gastroenterology of the Second Affiliated Hospital, Zhejiang University School of Medicine, Hangzhou, China; ^2^The Cancer Center of the Second Affiliated Hospital, Zhejiang University School of Medicine, Hangzhou, China; ^3^Collaborative Innovation Center for Diagnosis and Treatment of Infectious Diseases, Hangzhou, China; ^4^Department of Molecular Genetics, University of Toronto, Toronto, ON, Canada

**Keywords:** cell migration, filamin A, Hsp90, NudC-L279P, protein stability

## Abstract

Filamin A, the first discovered non-muscle actin filament cross-linking protein, plays a crucial role in regulating cell migration that participates in diverse cellular and developmental processes. However, the regulatory mechanism of filamin A stability remains unclear. Here, we find that nuclear distribution gene C (NudC), a cochaperone of heat shock protein 90 (Hsp90), is required to stabilize filamin A in mammalian cells. Immunoprecipitation-mass spectrometry and western blotting analyses reveal that NudC interacts with filamin A. Overexpression of human NudC-L279P (an evolutionarily conserved mutation in NudC that impairs its chaperone activity) not only decreases the protein level of filamin A but also results in actin disorganization and the suppression of cell migration. Ectopic expression of filamin A is able to reverse these defects induced by the overexpression of NudC-L279P. Furthermore, Hsp90 forms a complex with filamin A. The inhibition of Hsp90 ATPase activity by either geldanamycin or radicicol decreases the protein stability of filamin A. In addition, ectopic expression of Hsp90 efficiently restores NudC-L279P overexpression-induced protein stability and functional defects of filamin A. Taken together, these data suggest NudC L279P mutation destabilizes filamin A by inhibiting the Hsp90 chaperoning pathway and suppresses cell migration.

## Introduction

Cell migration, a highly integrated multistep process, plays a critical role in diverse cellular and developmental processes, including the inflammatory response, tissue repair, embryogenesis, and cancer metastasis (Ridley et al., 2003; George et al., 2013). Accumulating evidence indicates that the dynamic actin cytoskeleton spatially and temporally regulates cell migration (Tang and Gerlach, 2017). In general, cell migration is initiated by the formation of actin-based plasma membrane protrusions (termed lamellipodia and filopodia) at the leading edge of migrating cells (Lauffenburger and Horwitz, 1996; Small et al., 2002; Ridley et al., 2003; Machesky, 2008). There exists a dynamically remodeling of branched actin filament network in lamellipodia, which is tightly regulated by several actin regulators, including actin-related protein 2/3 (Arp2/3), cofilin, profilin, and filamins (Blanchoin et al., 2000; Cameron et al., 2000; Skau and Waterman, 2015).

Filamin A (also known as Filamin 1 and ABP-280) was the non-muscle actin cross-linking protein, and it is a member of the filamin family that includes filamin A, filamin B, and C (Kesner et al., 2010). Filamin A is widely expressed and plays an important role in cell migration regulation (Stossel et al., 2001; Feng and Walsh, 2004; Razinia et al., 2012; Kircher et al., 2015; Bandaru et al., 2019). Mutations in filamin A are associated with a range of human disorders termed filaminopathies A, including periventricular heterotopia, developmental regression, and West syndrome in males (Fox et al., 1998; Robertson et al., 2003; Zenker et al., 2004; Kyndt et al., 2007; Nurden et al., 2011). Filamin A often forms a non-covalent V-shaped homodimer composed of an N-terminal actin-binding domain (ABD) followed by 24 tandem immunoglobulin-like domains, the last of which mediates its dimerization (Stossel et al., 2001; Zhou et al., 2010). Filamin A crosslinks actin filaments (F-actin) into an isotropic, high-angle orthogonal branching, then sequentially polymerizes F-actin into the tightly organized orthogonal networks (Cukier et al., 2007; Kim and McCulloch, 2011; Kumar et al., 2019). Previous studies have shown that the activity of filamin A is regulated by phosphorylation to cross-link actin (Chen and Stracher, 1989; Schaefer et al., 2012; Hammer et al., 2013; Li et al., 2015; Sato et al., 2016). Recently, a series of studies indicate that Asb2 (ankyrin repeat-containing protein with a suppressor of cytokine signaling box 2), a specific subunit of CRL5 (Cullin 5-RING E3 ubiquitin ligases) is involved in proteasomal degradation of filamin A (Heuze et al., 2008; Razinia et al., 2011, 2013; Lamsoul et al., 2013; Spinner et al., 2015), however, the regulation of filamin A stability remains poorly understood.

Nuclear distribution gene C (NudC) is evolutionally conserved from yeast to human; it was first identified in the filamentous fungus *Aspergillus nidulans* as an upstream factor of NudF (an homolog of human LIS1, a key regulator of dynein) that could regulate nuclear movement (Osmani et al., 1990; Zhu et al., 2010; Fu et al., 2016; Jheng et al., 2018). NudC contains a core domain of p23 (p23 domain) (Zheng et al., 2011; Fu et al., 2016). P23 protein is a main cochaperone of Hsp90 (heat shock protein 90) participating in promoting the folding of various client proteins (Felts and Toft, 2003; Cox and Johnson, 2011). Emerging studies indicate that NudC may enhance the folding of their client proteins by itself chaperoning activity or functioning as an Hsp90 cochaperone by modulating Hsp90 ATPase activity (Zhu et al., 2010; Zheng et al., 2011; Li et al., 2012; Taipale et al., 2014; Zhang et al., 2016; Schopf et al., 2017; Dean and Johnson, 2021). Our recent study has shown that NudC is required to regulate actin dynamics and cell migration by stabilizing cofilin 1 in an Hsp90-independent manner (Zhang et al., 2016). We also found that NudC-L279P (a conserved mutation in human NudC to Leu^146^ in *Aspergillus* that leads to reduced NudF) impairs NudC itself and Hsp90 chaperone function (Zhu et al., 2010). NudC-L279P overexpression leads to reduced LIS1, a key regulator of cell migration, in an Hsp90-dependent manner (Zhu et al., 2010). However, the underlying mechanism of mammalian NudC in cell migration regulation remains unclear.

Here, we provide evidence that NudC stabilizes filamin A. Our data show that NudC interacts with filamin A. Overexpression of NudC L279P destabilizes filamin A and suppresses cell migration. Ectopic expression of Hsp90 reverses the instability of filamin A and phenotype defects caused by NudC-L279P overexpression. Thus, we propose that NudC L279P mutation destabilizes filamin A by inhibiting the Hsp90-mediated chaperoning pathway, providing a previously undescribed mechanism crucial for filamin A stability regulation.

## Materials and Methods

### Plasmids and Oligonucleotides

Human *GFP-NudC*, *GFP-NudC-L279P*, *Flag-NudC* and *Myc-Hsp90* were constructed as described previously (Yang et al., 2010; Zhu et al., 2010; Zhang et al., 2016). *Myc-filamin A* vector was a kind gift from John Blenis. Full-length human *LIS1* cloned by RT-PCR from RPE-1 cells was inserted into pcDNA3.1 (Clontech). All of these constructs were confirmed by DNA sequencing.

All siRNAs were synthesized by GenePharma. The sequences of the sense strands of the siRNA duplexes are as follows:

*NudC-1*: 5′-GAAGGGATGGCAGAGAAGC-3′ (Zhang et al., 2016);
*NudC-2*: 5′-AACACCTTCTTCAGCTTCCTT-3′ (Zhang et al., 2016);
*Filamin A*: 5′-CCAACAAGGUCAAAGUAUATT-3′ (Urra et al., 2018);
*LIS1*: 5′-CGGACAAGTAGAATAAATG-3′ (Yang et al., 2010).

### Cell Culture and Transfections

RPE-1 cells and AGS cells were maintained in Dulbecco’s modified Eagle’s medium/Ham’s F-12 medium (DMEM/F12, Corning) containing 10% fetal bovine serum (FBS, PAA Laboratories) at 37°C in 5% CO_2_. HeLa and HEK-293T cells were cultured in Dulbecco’s Modified Eagle’s Medium (DMEM, Corning) with 10% FBS at 37°C in 5% CO_2_. Plasmid transfection was performed using Lipofectamine 2000 (Invitrogen), and the synthetic oligonucleotides were transfected using Lipofectamine RNAiMAX (Invitrogen) according to manufacturer’s instructions.

### Preparation of Lentivirus and Construction of Stable Cell Lines

Lentiviruses were prepared as previously described (Lu et al., 2017). In brief, HEK-293T cells were transfected with the viral packaging constructs and the GFP, GFP-NudC or GFP-NudC-L279P vectors. The viral medium was collected 48 h post-transfection, filtered and mixed with fresh culture medium containing 10% FBS to infect the cells for 48 h. Infected cells were treated with 2 μg/ml puromycin for 72 h to select the plasmid-containing cells. Finally, the cell lines stably expressing the indicated proteins were identified by western blotting.

### Drug Treatments

Geldanamycin (GA, Tocris) and radicicol (RA, Tocris) were stored in the dark at −20°C as stock solutions at 1.78 mM in dimethylsulfoxide (DMSO, Sangon) and ethanol, respectively. Cells were treated with either GA or RA at the concentrations as described in the text for different times. MG132 (Millipore) was stored at −20°C as a stock solution at 5 mM in DMSO. Cells were treated with MG132 (5 μM) for 2 h. For cycloheximide (CHX, Sigma-Aldrich) chase analysis, 100 μg/ml CHX was used for the indicated times as described in the text.

### Antibodies

For western blotting analysis, antibodies against filamin A (1:1,000, 67133-1-Ig, Diagbio), Hsp90 (1:1,000, 13171-1-AP, Proteintech), NudC (1:1,000, 10681-1-AP, Proteintech), LIS1 (1:1,000, a12643, Proteintech), GAPDH (1:2,000, 60004-1-Ig, Proteintech), cofilin 1 (1:1,000, 10960-1-AP, Proteintech), NudCL (1:1,000, 11764-1-AP, Proteintech), Flag (1:1,000, AF519, Beyotime Biotechnology), and c-Myc (1:1,000, sc-40, Santa Cruz) were acquired commercially. Anti-NudCL2 antibody was generated as previously described (Yang et al., 2010). For immunofluorescence, antibody against filamin A (1:200, 67133-1-Ig, Diagbio) was used. The secondary antibodies for immunofluorescence analyses were Alexa Fluor 488-, 568-, and 647-conjugated anti-rabbit or anti-mouse IgG (1:500, Invitrogen). Goat anti-mouse or anti-rabbit secondary antibodies (1:5,000, LI-COR) conjugated with either Alexa Fluor 680 or IRDye 800 were used for western blotting.

### Immunoprecipitation and Western Blotting

Immunoprecipitation (IP) was performed as previously described (Li et al., 2019). In brief, cell extracts were generated in TBSN buffer (20 mM Tris [pH 8.0], 150 mM NaCl, 0.5% Non-idet P-40, 5 mM EGTA, 1.5 mM EDTA, 0.5 mM Na_3_VO_4_, 20 mM p-nitrophenyl phosphate) supplemented with protease inhibitors and subjected to coimmunoprecipitation (Co-IP) analysis with the indicated antibodies. Western blotting analyses were performed with the indicated antibodies and analyzed using the LI-COR Odyssey (LI-COR) system.

### Immunofluorescence Staining

Cells were grown on coverslips and fixed for 15 min with 4% paraformaldehyde in phosphate-buffered saline (PBS) at room temperature and incubated with primary antibodies for 2 h and secondary antibodies for 1 h at room temperature. Rhodamine-phalloidin (P1951, SignaGen) and 4,6-diamidino-2-phenylindole (DAPI, Sigma) were used to visualize F-actin and DNA, respectively. The images were acquired using a 63 × oil immersion objective (Zeiss, LSM880).

### Quantitative Real-Time RT-PCR

Quantitative RT-PCR analyses for *filamin A* were performed using a Bio-Rad CFX-Touch System with HiScript Q RT SuperMix (Vazyme). All of the reactions were performed at least three times. *GAPDH* was used as an internal control. The primers used to amplify the target *filamin A* were as follows:

Forward: 5′-ATCTTTACGGCAGGAGCTGG-3′;
Reverse: 5′-CTGGTAGCTGCAGCGGTATG-3′.

### Cell Tracking

Time-lapse video microscopy was used to track cell migration. The images were captured at 5-s intervals for 700 cycles with an LSM880 confocal microscope (Zeiss). The videos were further analyzed using Imaris 9.1.2 software.

### Kymography Analysis

For kymography analysis, phase-contrast time-lapse sequences were obtained using a 63 × oil immersion objective on an LSM880 Zeiss confocal microscope. Movies were recorded for 10-15 min at a rate of one frame every 3 s. Kymographs were produced and analyzed by ImageJ software (NIH). The quantitative analysis of kymographs was performed as previously described (Bear et al., 2002).

### Scratch Wound Experiment

Scratch wound assays were performed as described previously (Feng et al., 2017). In brief, cells were plated into 30-mm dishes and incubated at 37°C in 5% CO_2_ to establish a confluent monolayer. Then, cells were treated with serum-free medium for 12 h and scratched with a sterile pipette tip. Cellular debris was removed by washing the plate with PBS, and then fresh medium containing 1% FBS was added. Representative images of cells were taken at the indicated time points using a light microscope (Olympus, IX81). For lamellipodia formation assays, the cells were fixed and stained after 3 h of scratching.

### Transwell Assays

Transwell assays were performed as described previously (Chen et al., 2020). In brief, transwell inserts (8-μm pore, Corning) were placed into 24-well plates. A 200 μl suspension containing 80, 000 cells in medium with 1% FBS was placed in the upper chamber of a transwell apparatus, and 700 μl medium containing 20% FBS was added in the lower chamber. After 10 h of incubation at 37°C in 5% CO_2_, cells were fixed in 4% paraformaldehyde in PBS for 15 min and stained with 0.2% crystal violet for 20 min. Cells on the undersides of the filters were photographed using a microscope (20 × magnification). Five random fields were analyzed for each chamber and scored using ImageJ software.

### Cell Spreading Assay

Cell spreading assays were performed as described previously (Zhang et al., 2016). Briefly, cells were suspended in serum-free medium after trypsinization and incubated at 37°C in 5% CO_2_ for 1 h. Cells were reseeded on fibronectin (10 mg/ml)-coated plates. After 1 h, cells were washed with PBS and fixed with 4% paraformaldehyde in PBS. Photographs were taken using an LSM880 confocal microscope.

### Scanning Electron Microscope Analysis

RPE-1 cells grown on fibronectin (10 mg/ml)-coated glass coverslips were treated with extraction buffer (1% Triton X-100, 2% PEG [MW 35 kDa], 100 mM PIPES [free acid, pH 6.9], 1 mM MgCl2 and 1 mM EGTA, 2 μM phalloidin), washed with PBS, and fixed with 2% glutaraldehyde and processed for scanning electron microscope analysis as described previously (Nova NanoSEM 450 scanning electron microscope) (Nakamura, 2001).

### LC-MS/MS Analysis and Database Searching

Liquid chromatography/tandem mass spectrometry (LC-MS/MS) analysis was performed as described previously (Zhu et al., 2010). Briefly, HeLa cell lysates were incubated with anti-NudC antibodies. The proteins coimmunoprecipitated by antibodies were subjected to trypsin digestion, and the recovered peptide mixtures were separated by reversed-phase HPLC followed by tandem mass analysis at the Reach Center for Proteome Analysis, Shanghai Institutes of Biological Sciences. The peak lists of all acquired MS/MS spectra were generated by BioWorks software and then automatically searched against the human International Protein Index protein sequence database (version 3.36) using the SEQUEST algorithm (Deng et al., 2010).

### Statistics

Data are representative of at least three independent experiments. Means and standard deviations (SD) were calculated and are shown in the graphs. Student’s *t-*test was used to determine statistically significant differences between two groups.

## Results

### Overexpression of NudC-L279P Destabilizes Filamin A

**FIGURE 1:**
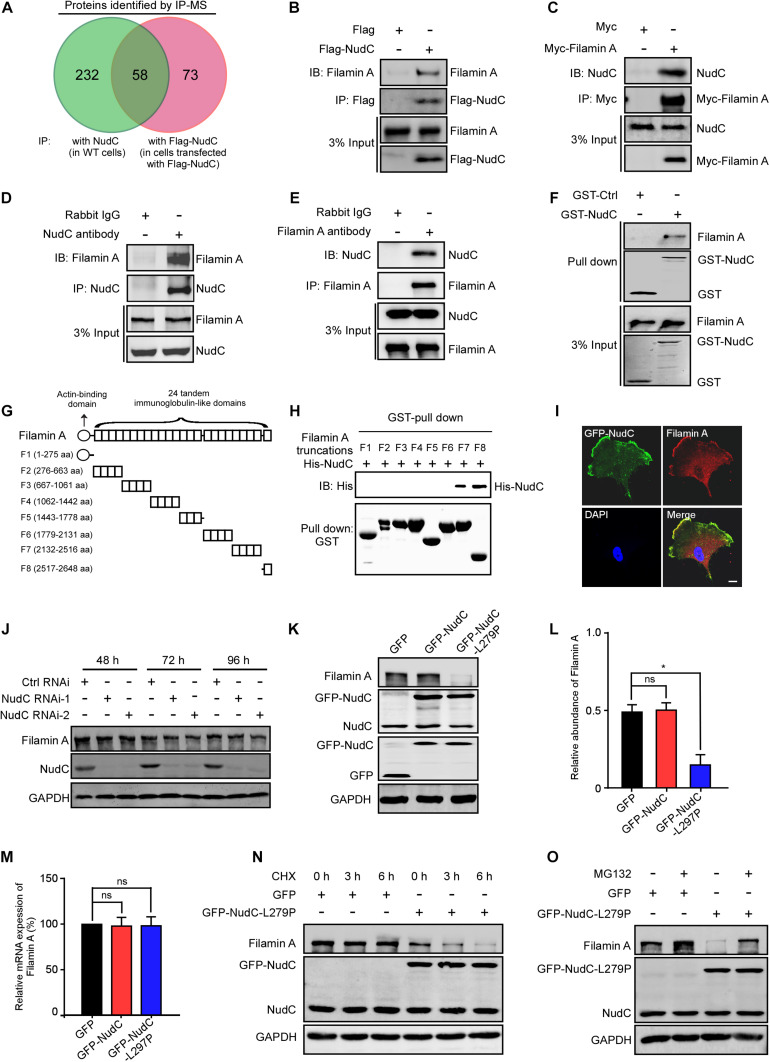
L279P mutation of NudC destabilizes filamin A. **(A)** Venn diagram showing the overlap between two sets of interactors identified by IP (with anti-NudC or anti-Flag antibodies) coupled with mass spectrometry in HeLa cells. **(B,C)** RPE-1 cells transfected with either Flag-NudC or Myc-Filamin A were subjected to IP with anti-Flag or anti-Myc antibodies and western blotting analysis, respectively. 3% of total input is shown. **(D,E)** Total lysates of RPE-1 cells were immunoprecipitated with the indicated antibodies or IgGs and processed for western blotting. 3% of total input is shown. **(F)** Purified GST or GST-NudC protein was incubated with RPE-1 cell lysates and subjected to immunoblotting with anti-filaminA antibody. 3% of total input is shown. GST and GST-NudC input were stained with Coomassie brilliant blue. **(G)** Summary of filamin A truncation mutants. **(H)** The indicated fragments of GST-tagged filamin A and His-NudC proteins were purified. Fragments were incubated with His-NudC and subjected to immunoblotting with the indicated antibodies. **(I)** Cells transfected with GFP-NudC were fixed and stained with anti-filamin A antibody. DNA was visualized with DAPI. Bar, 10 μm. **(J)** RPE-1 cells transfected with *NudC* RNAi-1 or -2 were harvested at various times and subjected to western blotting with anti-NudC and anti-filamin A antibodies. GAPDH, a loading control. **(K)** RPE-1 cells stably expressing GFP, GFP-NudC, or GFP-NudC-L279P were subjected to western blotting analysis by using anti-GFP, -NudC and -filamin A antibodies. GAPDH, a loading control. **(L)** Relative protein levels of filamin A compared to GAPDH in panels **(G)** were measured using ImageJ software. **(M)** Quantitative RT-PCR analysis of filamin A mRNA in RPE-1 cells transfected with the indicated vectors. GAPDH, an internal control. **(N)** Cells transfected with either GFP or GFP-NudC-L279P were treated with 100 μg/ml cycloheximide for different times, harvested, and subjected to western blotting analysis using indicated antibodies. GAPDH, a loading control. **(O)** Cells transfected with either GFP or GFP-NudC-L279P were treated with either 5 μM MG132 or DMSO, lysed, and subjected to western blotting using the indicated antibodies. GAPDH, a loading control. Quantitative data are presented as the means ± SD (at least three independent experiments). *P < 0.05 and ns, not significant (*P* > 0.05). Student’s *t*-test.

To identify the candidate proteins participating in NudC-mediated cell migration, we performed immunoprecipitation (IP) analysis with endogenous NudC followed by mass spectrometry (MS) in HeLa cells and found that approximately three hundred proteins appeared to associate with NudC (Supplementary Table 1, ranked based on relative abundance). When comparing these proteins with our previous interactome data for Flag-NudC (Zhu et al., 2010; Supplementary Table 2, ranked based on relative abundance), we found 58 overlapping proteins (Figure 1A and Supplementary Table 3). Notably, we found that the relative abundance of filamin A is the highest in both interaction datasets (Supplementary Tables 1, 2). IP analysis confirmed that filamin A interacted with Flag-NudC in RPE-1 cells (Figure 1B). NudC formed a complex with Myc-filamin A (Figure 1C). In addition, the results of western blotting following IP and GST pull-down assays verified the interaction of NudC and filamin A in RPE-1 cells (Figures 1D–F). To examine whether the direct interaction exists between NudC and filamin A, we analyzed the amino acid sequence of filamin A and constructed 8 truncations according to the protein domains in filamin A. Then we expressed and purified GST-filamin A fragments and His-NudC and carried out GST pull-down analysis. The results showed that F7 and F8 of filamin A appeared to interact with His-NudC (Figures 1G,H), suggesting that there is a direct interaction between NudC with both the 20-23 repeats (2132-2516 aa) and with repeat 24 (2517-2648 aa) of filamin A. Furthermore, immunofluorescence results revealed that GFP-NudC appeared to colocalize with filamin A throughout the cytoplasm of RPE-1 cells, especially at the leading edge of motile cells (Figure 1I).

Previous data from our laboratory and others suggest that NudC is involved in the regulation of protein stability (Zhu et al., 2010; Zhang et al., 2016). To test whether NudC regulates the stability of filamin A, we used two siRNA oligos targeting two different regions of *NudC* mRNA (NudC RNAi-1 and NudC RNAi-2). The filamin A protein level showed no obvious change in NudC-depleted cells compared to control cells (Figure 1J and Supplementary Figure 1A). Our previous study demonstrated that the L279P mutation of NudC impairs its chaperone activity to affect protein stability. Thus, we explored whether the L279P mutation of NudC influenced the protein stability of filamin A. We constructed lentiviral vectors containing GFP, GFP-NudC, or GFP-NudC-L279P to establish cell lines stably expressing the above proteins in RPE-1, HeLa and AGS cells. Western blotting revealed that GFP and GFP-fusion proteins were successfully expressed (Figure 1K). We found that the protein level of filamin A was substantially decreased in cells stably expressing NudC-L279P compared to the control cells (Figures 1K,L and Supplementary Figure 1B), whereas its mRNA level was not significantly changed based on RT-PCR analysis (Figure 1M). CHX chase analysis revealed that the degradation rate of filamin A was faster in cells stably expressing GFP-NudC-L279P compared to control cells (Figure 1N). Moreover, treating cells with the proteasome inhibitor MG132 inhibited the degradation of filamin A in cells stably expressing GFP-NudC-L279P (Figure 1O), suggesting that the ubiquitin-proteasome pathway is involved in filamin A degradation. Taken together, these data strongly suggest that the NudC L279P mutation destabilizes filamin A.

### Overexpression of NudC-L279P Inhibits Cell Migration

**FIGURE 2:**
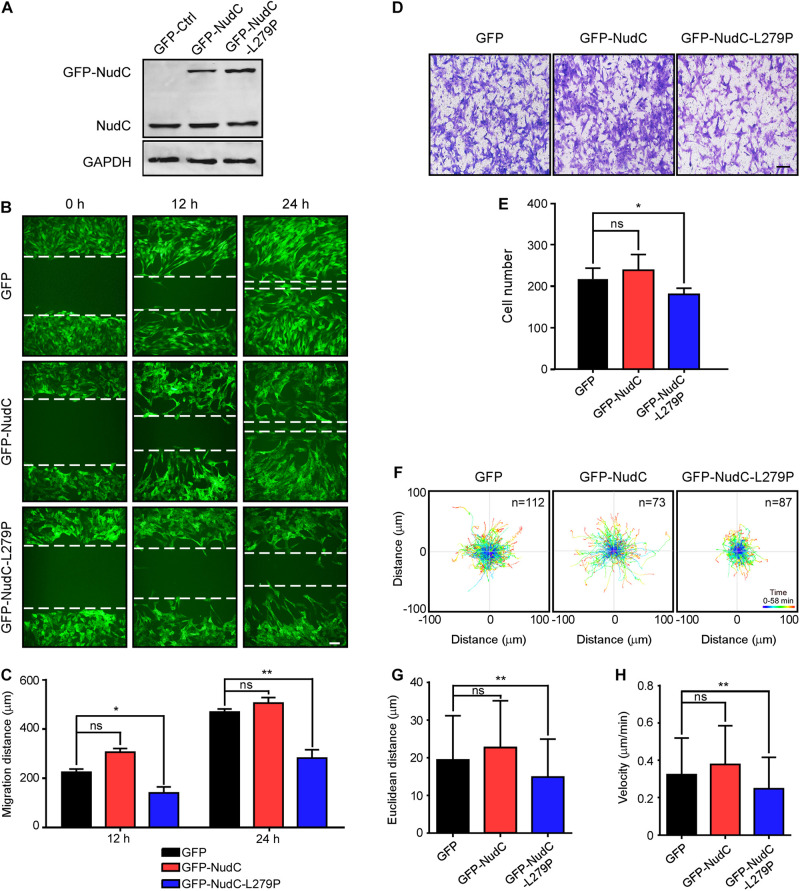
Overexpression of NudC-L279P inhibits cell migration in RPE-1 cells. RPE-1 cells stably expressing GFP, GFP-NudC, or GFP-NudC-L279P were subjected to the following analyses. **(A)** Western blotting analysis of the expression of GFP, GFP-NudC, or GFP-NudC-L279P. GAPDH, a loading control. **(B,C)** Scratch wound assays displayed cell migration at the different time points. Cells expressing GFP signals were monitored with fluorescence microscopy. Dashed lines indicate the approximate line of wound edges. The distance between the two edge lines was measured by ImageJ software. Scale bar, 100 μm. **(D,E)** Transwell migration assays were performed to detect cell motility. Cells that migrated to the undersides of the filters were stained with 0.2% crystal violet and monitored with DIC (differential interference contrast) microscopy. The number of migrated cells per transwell was counted. **(F–H)** The migration track of individual cells was traced using Imaris 9.1.2 software. Representative cell migration tracks are shown. Euclidean distance and migration velocity were analyzed with Imaris 9.1.2 software. Scale bar, 100 μm. Quantitative data are presented as the means ± SD (at least three independent experiments). n, the sample size. *P < 0.05; **P < 0.01; and ns, not significant (*P* > 0.05). Student’s *t*-test.

Previous studies have shown that filamin A plays an important role in cell migration regulation (Cunningham et al., 1992; Fox et al., 1998; Sutherland-Smith, 2011; Jacquemet et al., 2013; Bandaru et al., 2019). The downregulation of filamin A inhibits cell migration in mammalian cells (Cunningham et al., 1992; Jacquemet et al., 2013; Bandaru et al., 2019), which is consistent with our data (Supplementary Figures 2, 3). Given that NudC-L279P overexpression led to filamin A instability (Figure 1), we tested whether the L279P mutation of NudC has an effect on cell migration. Scratch wound assays revealed that NudC-L279P overexpression significantly suppressed cell migration compared to the control in RPE-1 cells (Figures 2A–C). Transwell migration assays also showed that the L279P mutation of NudC decreased RPE-1 cell migration (Figures 2D,E). Moreover, tracing the migratory path of live cells by time-lapse microscopy revealed that NudC-L279P overexpression decreased the speed of RPE-1 cell motility (Figures 2F–H and Supplementary Movies 1–3). Similar results were also found in AGS cells (Supplementary Figure 4). Thus, these data suggest that L279P mutation of NudC suppresses cell migration.

### Overexpression of NudC-L279P Impairs Actin Dynamics

**FIGURE 3:**
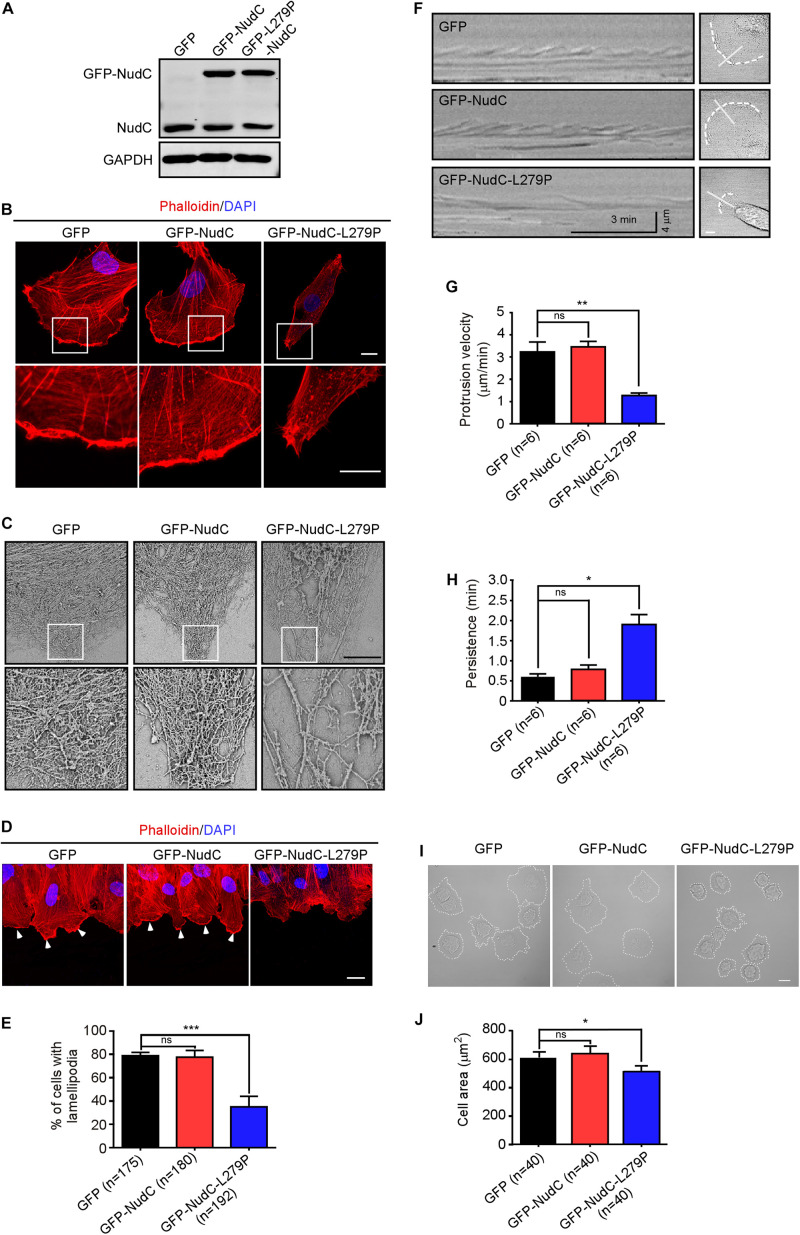
Overexpression of NudC-L279P impairs actin dynamics. RPE-1 cells infected with lentiviruses expressing GFP, GFP-NudC, or GFP-NudC-L279P were subjected to the following analyses. **(A)** Western blotting analysis of the expression of the indicated proteins. GAPDH, a loading control. **(B)** Cells were fixed and stained with phalloidin. DNA was visualized with DAPI. Images were captured using an immunofluorescence microscope. Scale bar, 10 μm. Higher magnifications of the boxed regions are displayed. **(C)** Structural organization of lamellipodia is shown with scanning electron microscopy. Scale bar, 5 μm. Higher magnifications of the boxed regions are displayed. **(D,E)** Cells stably expressing the indicated proteins were fixed and stained with phalloidin after 3 h of scratching. DNA was visualized by DAPI. Scale bar, 20 μm. The lamellipodia at the leading edge of cells are indicated by arrowheads. Cells with lamellipodia were calculated. **(F–H)** A sequence of phase-contrast time-lapse images of the cells was obtained with an LSM880 confocal microscope. Kymographs were analyzed using MetaMorph software. The minimum intensity projection of a 250-frame movie (3 s per frame) is presented on the left. Pixel intensities along a one-pixel-wide line (white) were used to generate the kymograph presented on the right. Cells are outlined with dashed lines. **(I,J)** Cell spreading was detected using a phase contrast microscope. The areas of cell spreading are outlined by dashed lines and measured by ImageJ software (NIH). Scale bar, 10 μm. Quantitative data are presented as the means ± SD (at least three independent experiments). n, the sample size. *P < 0.05; **P < 0.01; ***P < 0.001; and ns, not significant (*P* > 0.05). Student’s *t*-test.

Given that filamin A regulates cell migration mainly by controlling dynamic actin cytoskeleton remodeling (Ohta et al., 1999; Nishita et al., 2006; Maceyka et al., 2008; Venkatareddy et al., 2011) and NudC L279P mutation destabilizes filamin A (Figure 1), we tested whether overexpression of NudC-L279P would affect the structure and function of the actin cytoskeleton. Immunostaining assay using fluorescent phalloidin-labeled F-actin showed that NudC-L279P overexpression destroyed dynamic actin networks in lamellipodia at the leading edge of cells (Figures 3A,B). Scanning electron microscopy also revealed that the L279P mutation impaired cross-linked actin networks and caused an increase in the unbranched actin filaments in lamellipodia (Figure 3C). Furthermore, immunostaining data showed that NudC-L279P overexpression significantly decreased lamellipodia formation at the leading edge after scratching (Figures 3D,E). Kymographs of lamellipodial protrusion showed that the NudC L279P mutation caused a significant decrease in protrusion velocity with a concomitant increase in protrusion persistence (Figures 3F–H). Moreover, phase contrast images showed that NudC L279P suppressed cell spreading (Figures 3I,J). Collectively, these data suggest that the NudC L279P mutation impairs actin dynamics.

### Enforced Expression of Filamin A Reverses the Phenotypes Induced by NudC-L279P Overexpression

**FIGURE 4:**
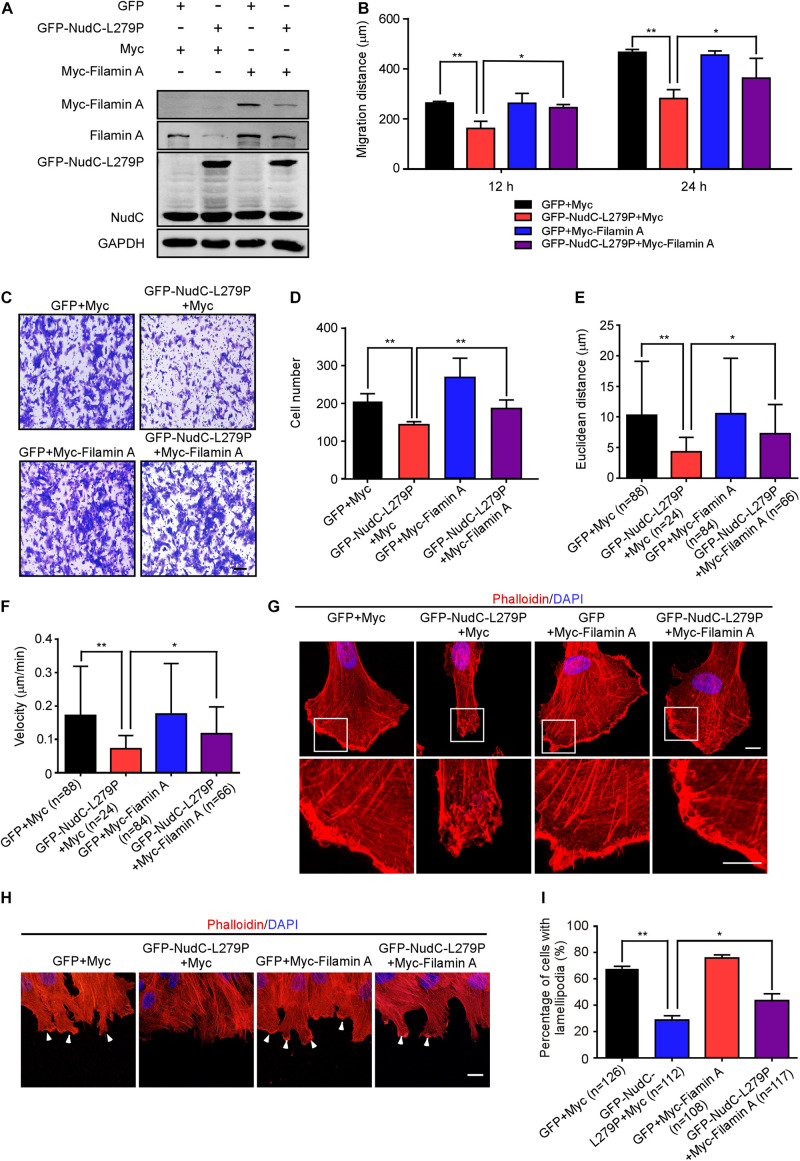
Enforced expression of filamin A reverses the defects caused by NudC-L279P overexpression. RPE-1 cells stably overexpressing GFP or GFP-NudC-L279P were transfected with Myc or Myc-filamin A and then subjected to the following analyses. **(A)** Western blotting analysis of the expression of the indicated proteins. GAPDH, a loading control. **(B)** Scratch wound assays detected cell migration at the different time points. The scratch closure was monitored with fluorescence microscopy. The distance between the two edge lines was measured using ImageJ software. **(C,D)** Transwell migration assays were performed to detect cell migration. Cells that migrated to the undersides of the filters were stained with 0.2% crystal violet and monitored with DIC microscopy. The number of migrated cells per transwell was counted. Scale bar, 100 μm. **(E,F)** The migration tracks of individual cells were traced by Imaris 9.1.2 software. Euclidean distance and migration velocity were analyzed with Imaris 9.1.2 software. **(G)** Cells were fixed and stained with phalloidin. DNA was visualized with DAPI. Images were captured by immunofluorescence microscopy. Scale bar, 10 μm. Higher magnifications of the boxed regions are displayed. **(H,I)** Cells were fixed and stained with phalloidin after 3 h of scratching. DNA was visualized by DAPI. Scale bar, 5 μm. The lamellipodia at the leading edge of cells are pointed by arrowheads. Cells with lamellipodia were counted. Quantitative data are presented as the means ± SD (at least three independent experiments). n, the sample size. *P < 0.05 and **P < 0.01. Student’s *t*-test.

Given that NudC-L279P overexpression destabilizes filamin A and results in defects in actin dynamics and cell migration, we investigated whether filamin A participates in NudC-mediated cell migration. Western blotting revealed that Myc-filamin A was successfully expressed in cells stably overexpressing GFP or GFP-NudC-L279P (Figure 4A). Scratch wound, transwell and live cell migration assays showed that ectopic filamin A expression significantly reversed the defects of cell migration in cells that stably expressing NudC-L279P (Figures 4B–F and Supplementary Figure 5). Furthermore, immunofluorescence results revealed that the disorganization of actin networks in lamellipodia and the decrease in lamellipodia formation in cells stably expressing NudC-L279P were partially reversed by filamin A overexpression (Figures 4G–I). Collectively, these data suggest that filamin A is involved in NudC-mediated cell migration regulation.

### Hsp90 Binds to and Stabilizes Filamin A

**FIGURE 5:**
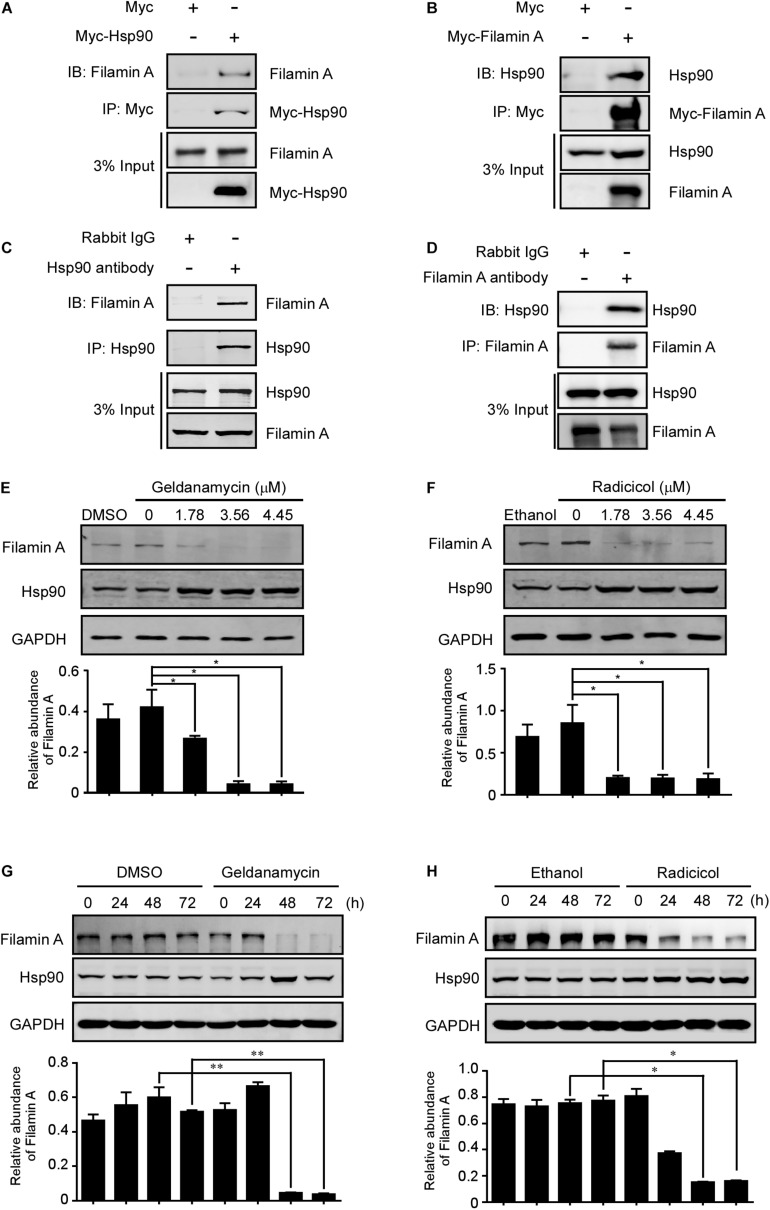
Hsp90 binds to and stabilizes filamin A. **(A,B)** RPE-1 cells transfected with either Myc-Hsp90 or -Filamin A were subjected to IP and western blotting using the indicated antibodies. 3% of total input is shown. **(C,D)** Total lysates of RPE-1 cells were immunoprecipitated with anti-Hsp90, -filamin A, or -IgG antibodies. Then, the samples were subjected to western blotting analysis with the indicated antibodies. 3% of total input is shown. **(E,F)** RPE-1 cells were treated with different concentrations of GA or RA for 48 h and subjected to western blotting analyses with the indicated antibodies as shown. Relative protein levels of filamin A compared to GAPDH were measured using ImageJ software and are shown at the bottom. **(G,H)** RPE-1 cells were treated with 1.78 μM GA or RA for different times and then processed for western blotting analyses with the indicated antibodies. The relative abundances of filamin A compared to GAPDH were measured using ImageJ software and are shown at the bottom. Quantitative data are presented as the means ± SD (at least three independent experiments). *P < 0.05 and **P < 0.01. Student’s *t*-test.

Previous data from our group and others suggest that NudC may be involved in the regulation of protein stability through Hsp90-dependent and Hsp90-independent pathways (Zhu et al., 2010; Fu et al., 2016; Zhang et al., 2016). To test whether Hsp90 is involved in the regulation of filamin A stability, we performed IP experiments in RPE-1 cells transfected with Flag-Hsp90 or Myc-filamin A and found that an interaction between filamin A and Hsp90 (Figures 5A,B). In addition, western blotting following IP verified the association of endogenous Hsp90 with filamin A (Figures 5C,D). Furthermore, we employed GA and RA, which inhibits Hsp90 ATPase activity and leads to proteasomal degradation of Hsp90 client proteins (Kamal et al., 2004; Pearl and Prodromou, 2006). Western blotting indicated that the inhibition of Hsp90 chaperone activity destabilized filamin A instability in a dose- and time-dependent manner in mammalian cells (Figures 5E–H and Supplementary Figure 6). Taken together, these data suggest that Hsp90 is involved in the regulation of filamin A protein stability.

### Ectopic Expression of Hsp90 Reverses the Defects Induced by NudC-L279P Overexpression

**FIGURE 6:**
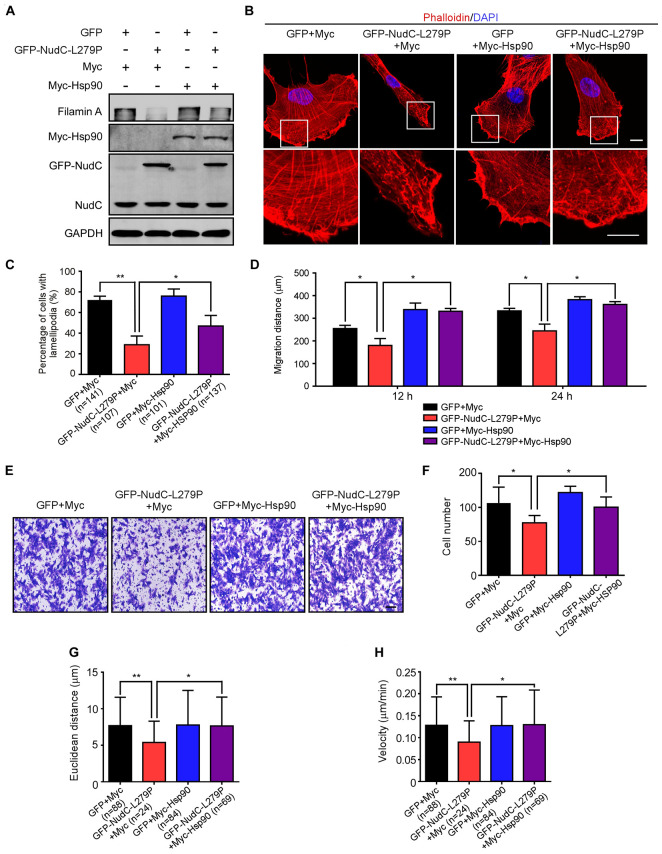
Ectopic expression of Hsp90 reverses the defects induced by NudC-L279P overexpression. RPE-1 cells stably overexpressing GFP or GFP-NudC-L279P were transfected with Myc or Myc-Hsp90 and then subjected to the following analyses. **(A)** Western blotting analysis of the expression of the indicated proteins. GAPDH, a loading control. **(B)** Cells were fixed and stained with phalloidin. DNA was visualized with DAPI. Images were captured by immunofluorescence microscopy. Scale bar, 10 μm. Higher magnifications of the boxed regions are displayed. **(C)** Cells were fixed and stained with phalloidin after 3 h of scratching. Cells with lamellipodia were counted. **(D)** Scratch wound assays detected cell motility. The distance of scratch closure was measured by ImageJ software. **(E,F)** Transwell migration assays were performed to detect cell motility. Cells that migrated to the undersides of the filters were stained with 0.2% crystal violet and monitored with DIC microscopy. The number of migrated cells per transwell was calculated. Scale bar, 100 μm. **(G,H)** The migration tracks of individual cells were traced by Imaris 9.1.2 software. Euclidean distance and migration velocity were analyzed with Imaris 9.1.2 software. Quantitative data are presented as the means ± SD (at least three independent experiments). n, the sample size. *P < 0.05 and **P < 0.01. Student’s *t*-test.

Because both overexpression of L279P and Hsp90 inhibition destabilizes filamin A and the NudC L279P mutation suppresses cell migration, we tested whether Hsp90 is involved in NudC-medicated cell migration via filamin A. We performed a series of rescue experiments and found that ectopic Hsp90 expression reversed the decrease of filamin A protein levels in both RPE-1 and AGS cells stably expressing NudC-L279P (Figure 6A and Supplementary Figure 7A). Immunofluorescence data showed that the defects in actin networks and lamellipodia formation induced by NudC-L279P overexpression were partially reversed by Hsp90 overexpression (Figures 6B,C). Furthermore, scratch wound, transwell, and live cell migration assays revealed that ectopic Hsp90 expression restored the defects induced by NudC-L279P overexpression (Figures 6D–H and Supplementary Figures 7B–G). Together, these data suggest that Hsp90 is involved in NudC-mediated cell migration via filamin A.

### NudCL and NudCL2 Are Involved in the Regulation of Filamin A Stability Coordinating With NudC

Vertebrate NudC has three principal homologs including NudC, NudC-like protein (NudCL), and NudC-like protein 2 (NudCL2) (Zhou et al., 2006; Yang et al., 2010; Zheng et al., 2011; Fu et al., 2016). All members of the NudC family share a conserved p23 domain and may function as Hsp90 cochaperone by modulating Hsp90 ATPase activity to enhance the folding of their client proteins (Zhu et al., 2010; Zheng et al., 2011; Taipale et al., 2014; Yang et al., 2019). In this study, our results displayed that overexpression of NudC L279P, but not overexpression of NudC or single knockdown, causes a decrease in filamin A protein level (Figure 1). Interestingly, our recent protein interactome analysis of NudCL2 showed that NudCL2 is able to form a complex with filamin A, but its depletion has no obvious effect on the protein level of filamin A (Chen et al., 2020). These data prompted us to suppose that NudCL2 and/or NudCL may be involved in the regulation of filamin A stability coordinating with NudC. To address this hypothesis, we designed and carried out a series of experiments. Western blotting results showed that depletion of one member of NudC family has no obvious effect on filamin A protein level (Supplementary Figure 8A). However, double depletion, especially triple depletion of all NudC family resulted in an obvious decrease in filamin A protein level (Supplementary Figure 8A). Furthermore, scratch wound assays revealed that depletion of NudC or NudCL but not NudCL2 suppresses cell migration (Supplementary Figures 8B,C), consistenting with our previous studies (Zhang et al., 2016; Chen et al., 2020). It is notable that knockdown of any two or three NudC members led to a significant suppression in cell migration (Supplementary Figures 8B,C). Furthermore, Co-IP analyses showed that NudC, NudCL or NudCL2 was able to form a complex with filamin A and Hsp90 (Supplementary Figure 8D). Taken together, these data suggest that NudC, NudCL, and NudCL2 may synergistically regulate filamin A stability in mammalian cells (Supplementary Figure 8E).

### Ectopic Expression of LIS1 Is Able to Reverse Cell Migration Defects Caused by NudC-L279P Overexpression

Our previous study showed that the overexpression of human NudC-L279P results in a decrease in the protein level of LIS1 (Zhu et al., 2010). Here, we found that NudC-L279P overexpression destabilizes filamin A and suppresses cell migration. To address whether the suppression of cell migration caused by NudC-L279P overexpression also is mediated by LIS1, we carried out rescue assays in cells overexpressing NudC-L279P. The results showed that ectopic expression of LIS1 in RPE-1 cells was able to reverse the inhibition of cell migration caused by NudC-L279P overexpression (Supplementary Figure 9). The further data revealed that LIS1 depletion had no obvious effect on the stability of filamin A, and vice versa (Supplementary Figure 10). Together, these data suggest that LIS1 is involved in NudC-mediated the regulation of cell migration with filamin A.

## Discussion

Cell migration plays a central role in a wide variety of biological processes (Friedl and Wolf, 2010; Yilmaz and Christofori, 2010), however, the underlying regulatory mechanisms remain incompletely understood. Here, our data show that NudC forms a complex with filamin A. The overexpression of NudC-L279P causes filamin A instability, actin disorganization, and cell migration suppression in mammalian cells. Furthermore, our results show that ectopic Hsp90 expression reverses filamin A degradation and functional defects caused by NudC-L279P overexpression. Together, these data suggest NudC L279P mutation destabilizes filamin A by impairing Hsp90-mediated chaperoning pathway and suppresses cell migration.

Mammalian filamins are a family of actin cross-linking proteins including filamin A, filamin B, and C (Kesner et al., 2010). Previous studies have revealed that filamin A is phosphorylated by several kinases to regulate its ability to cross-link actin (Hammer et al., 2013; Li et al., 2015; Sato et al., 2016). Recent studies also showed that the stability of filamin A is negatively regulated by FILIP (filamin A interacting protein 1) and Asb2α (Nagano et al., 2002, 2004; Sato and Nagano, 2005; Heuze et al., 2008; Razinia et al., 2011, 2013; Lamsoul et al., 2013; Spinner et al., 2015; Métais et al., 2018). Asb2α is able to target filamin A and B for proteasomal degradation (Heuze et al., 2008; Razinia et al., 2011, 2013; Lamsoul et al., 2013; Spinner et al., 2015; Métais et al., 2018). Accumulating studies also indicate that the removal of filamin A and filamin B results in defects in migration and cell spreading (Sheen et al., 2002; Baldassarre et al., 2009, 2012). Here, we provide evidence that overexpression of NudC L279P but not its depletion destabilizes filamin A *via* the ubiquitin-proteasome pathway, which is reversed by exogenous expression of Hsp90 (Figures 1, 6). Furthermore, our results also show that NudCL or NudCL2 forms a complex with filamin A and Hsp90, and participates in the regulation of filamin A stability by coordinating with NudC (Supplementary Figure 8). NudC L279P mutant may act as a dominant negative mutation to destabilize filamin A stability. Together, these data suggest a molecular mechanism of the positive regulation of filamin A stability by NudC family.

Cell migration is a highly integrated multistep process, which is regulated by a series of key regulators (Blanchoin et al., 2000; Cameron et al., 2000; Friedl and Wolf, 2010; Yilmaz and Christofori, 2010; Skau and Waterman, 2015). Our previous data showed that depletion of NudC inhibits cell migration by decreasing the protein stability of cofilin 1 via Hsp90-independent pathway (Zhu et al., 2010; Fu et al., 2016). We also found that NudCL2, which is cloned and characterized by our group, is involved in the regulation of cell migration by comodulating the stability of myosin-9 and LIS1 through Hsp90 pathway (Chen et al., 2020). In this report, our data show that NudC is involved in the regulation of cell migration also by positively regulating filamin A stability through the Hsp90 pathway, which is coordinated by NudCL and NudCL2. Meanwhile, our data display that LIS1 also participates in NudC-mediated cell migration regulation. Ectopic expression of LIS1 is able to reverse cell migration defects caused by NudC-L279P overexpression (Supplementary Figure 9). Taken together, these studies suggest that NudC family plays an important role in cell migration regulation by stabilizing a series of client proteins.

## Data Availability Statement

The original contributions presented in the study are included in the article/Supplementary Material, further inquiries can be directed to the corresponding author/s.

## Author Contributions

ML and ZX designed the study, performed experiments, and wrote the manuscript. CZ designed the study and performed some experiments. CY, JF, YL, and WZ performed some of the experiments. WC, XX, XS, MY, and WL edited the manuscript. TZ and YY conceived the study, participated in its design, and contributed to manuscript writing. All authors contributed to the article and approved the submitted version.

## Conflict of Interest

The authors declare that the research was conducted in the absence of any commercial or financial relationships that could be construed as a potential conflict of interest.

## Supplementary Material

Supplementary Figure 1 L279P mutation of NudC destabilizes filamin A in AGS cells. **(A)** Cells transfected with *NudC* RNAi-1, -2 or control were harvested at the indicated times. The cell lysates were subjected to western blotting analysis with anti-NudC and anti-filamin A antibodies. GAPDH, a loading control. **(B)** Cells stably expressing GFP, GFP-NudC or GFP-NudC-L279P were subjected to western blotting analysis using the antibodies as shown. GAPDH, a loading control.

Supplementary Figure 2 Depletion of filamin A suppresses cell migration in RPE-1 cells. Cells were treated with *filamin A* RNAi or not were subjected to the following analyses. **(A)** Western blotting showed the expression of filamin A. GAPDH, a loading control. **(B,C)** Scratch wound assays revealed cell migration at the different time points. The scratch closure was recorded with DIC microscopy. Dashed lines were used to indicate the approximate line of wound edges. The distance between the two edge lines was measured by ImageJ software. **(D,E)** Transwell migration assays were carried out to detect cell motility. Cells that migrated to the undersides of the filters were stained with 0.2% crystal violet and monitored with DIC microscopy. The number of migrated cells per transwell was counted. **(F-H)** The migration tracks of individual cells were traced by Imaris 9.1.2 software. Representative cell migration tracks are shown. Euclidean distance and migration velocity were analyzed with Imaris 9.1.2 software. Scale bars, 100 μm. Quantitative data are presented as the means ± SD (at least three independent experiments). n, the sample size. ^∗^*P* < 0.05 and ^∗∗∗^*P* < 0.001. Student’s *t*-test.

Supplementary Figure 3 Downregulation of filamin A suppresses cell migration in AGS cells. Cells treated with *filamin A* RNAi or not were subjected to the following analyses. **(A)** Western blotting analysis showed the expression of filamin A. GAPDH, a loading control. **(B,C)** Scratch wound assays revealed cell migration at the different time points. The scratch closure was recorded with DIC microscopy. Dashed lines indicated the approximate line of wound edges. The distance between the two edge lines was measured by ImageJ software. **(D,E)** Transwell migration assays were performed to detect cell motility. Cells that migrated to the undersides of the filters were stained with 0.2% crystal violet and monitored with DIC microscopy. The number of migrated cells per transwell was counted. Scale bar, 50 μm. **(F-H)** The migration tracks of individual cells were traced by Imaris 9.1.2 software. Euclidean distance and migration velocity were analyzed with Imaris 9.1.2 software. Scale bars, 100 μm. Quantitative data are presented as the means ± SD (at least three independent experiments). n, the sample size. ^∗^*P* < 0.05 and ^∗∗^*P* < 0.01. Student’s *t*-test.

Supplementary Figure 4 Overexpression of NudC-L279P suppresses cell migration in AGS Cells. AGS cells stably overexpressing GFP, GFP-NudC, or GFP-NudC-L279P were subjected to following analyses. **(A)** Western blotting analysis of the expression levels of the indicated proteins. GAPDH, a loading control. **(B,C)** Scratch wound assays revealed cell migration at the different time points. The scratch closure was recorded with fluorescence microscopy. Dashed lines defined the approximate line of wound edges. The distance between the two edge lines was measured by ImageJ software. **(D,E)** Transwell migration assays were carried out to detect cell motility. Cells that migrated to the undersides of the filters were stained with 0.2% crystal violet and monitored with DIC microscopy. The number of migrated cells per transwell was counted. **(F-H)** The migration tracks of individual cells were traced by Imaris 9.1.2 software. Euclidean distance and migration velocity were analyzed with Imaris 9.1.2 software. Scale bars, 100 μm. Quantitative data are presented as the means ± SD (at least three independent experiments). n, the sample size. ^∗^*P* < 0.05; ^∗∗^*P* < 0.01; ^∗∗∗^*P* < 0.001; and ns, not significant (*P* > 0.05). Student’s *t*-test.

Supplementary Figure 5 Ectopic expression of filamin A reverses the defects of cell migration induced by NudC-L279P overexpression in AGS cells. Cells were transfected with the indicated vectors, and then subjected to the following analyses. **(A)** Western blotting analysis of the expression levels of the indicated proteins. GAPDH, a loading control. **(B,C)** Scratch wound assays revealed cell migration at the different time points. The scratch closure was monitored with fluorescence microscope. Dashed lines defined the approximate line of wound edges. The distance between the two edge lines was measured by ImageJ software. **(D,E)** Transwell migration assays were performed to detect cell motility. Cells that migrated to the undersides of the filters were stained with 0.2% crystal violet and monitored with DIC microscopy. The number of migrated cells per transwell was counted. **(F,G)** The migration tracks of individual cells were traced by Imaris 9.1.2 software. Euclidean distance and migration velocity were analyzed with Imaris 9.1.2 software. Scale bars, 100 μm. Quantitative data are presented as the means ± SD (at least three independent experiments). n, the sample size. ^∗^*P* < 0.05; ^∗∗^*P* < 0.01; and ^∗∗∗^*P* < 0.001. Student’s *t*-test.

Supplementary Figure 6 Inhibition of Hsp90 decreases the protein levels of filamin A in AGS cells. **(A,B)** AGS cells were incubated with different concentrations of GA or RA for 48 h, and then subjected to Western blotting analyses with the indicated antibodies. GAPDH, a loading control. Relative protein levels of filamin A compared to GAPDH were measured using ImageJ software and shown at the bottom. **(C,D)** AGS cells were incubated with 1.78 μM GA or RA for different times, and then processed for western blotting analyses. GAPDH, a loading control. Relative protein levels of filamin A compared to GAPDH are shown at the bottom. Quantitative data are presented as the means ± SD (at least three independent experiments). ^∗^*P* < 0.05; ^∗∗^*P* < 0.01; and ^∗∗∗^*P* < 0.001. Student’s *t*-test.

Supplementary Figure 7 Ectopic expression of Hsp90 reverses the defects in cell migration induced by NudC-L279P overexpression in AGS cells. Cells transfected with indicated vectors were subjected the following analyses. **(A)** Western blotting analysis of the proteins’ level with the indicated antibodies. GAPDH, a loading control. **(B,C)** Scratch wound assays indicated cell migration at the different time points. The scratch closure was monitored with fluorescence microscope. Dashed lines defined the approximate line of wound edges. The distance between the two edge lines was measured by ImageJ software. **(D,E)** Transwell migration assays were performed to detect cell motility. Cells that migrated to the underside of the filter were stained with 0.2% crystal violet and monitored with DIC microscopy. The number of migrated cells per transwell was plotted. **(F,G)** The migration tracks of individual cells were traced by Imaris 9.1.2 software. Euclidean distance and migration velocity were analyzed with Imaris 9.1.2 software. Scale bars, 100 μm. Quantitative data are presented as the means ± SD (at least three independent experiments). ^∗^*P* < 0.05. Student’s *t*-test.

Supplementary Figure 8 NudCL and NudCL2 are involved in the regulation of filamin A stability coordinating with NudC. **(A)** RPE-1 cells were treated with the indicated siRNAs were subjected to western blotting analysis using the antibodies as shown. GAPDH, the loading control. **(B)** Cells treated with the indicated siRNAs were subjected to scratch wound assays. The scratch closure was monitored with DIC microscopy. The distance between the two edge lines was measured using ImageJ software. Scale bar, 100 μm. **(C)** The distance between the two edge lines of scratch wound assays was measured by ImageJ software. Differences were statistically compared with the control. **(D)** Total lysates of cells were immunoprecipitated with the filamin A antibodies or IgGs and processed for western blotting. 3% of total input is shown. **(E)** Model for the role of NudC family in cell migration regulation. NudC, NudCL, and NudCL2 synergistically regulate filamin A stability and cell migration by the Hsp90 pathway. Scale bar, 100 μm. Quantitative data are presented as the means ± SD (at least three independent experiments). ^∗∗^*P* < 0.01; ^∗∗∗^*P* < 0.001; and ns, not significant (*P* > 0.05), Student’s *t*-test.

Supplementary Figure 9 Ectopic expression of LIS1 partially reverses the defects of cell migration induced by NudC-L279P overexpression. RPE-1 cells stably overexpressing GFP, GFP-NudC, or GFP-NudC-L279P were transfected with the indicated vectors, and then subjected to the following analyses. **(A)** Western blotting analysis of the proteins’ level with the indicated antibodies. GAPDH, a loading control. **(B,C)** Scratch wound assays revealed cell migration at the different time points. The scratch closure was monitored with fluorescence microscope. Dashed lines indicated the approximate line of wound edges. The distance between the two edge lines was measured by ImageJ software. **(D,E)** Transwell migration assays were performed to analyze cell motility. Cells that migrated to the underside of the filter were stained with 0.2% crystal violet and monitored with DIC microscopy. The number of migrated cells per transwell was counted. **(F,G)** The migration tracks of individual cells were traced by Imaris 9.1.2 software. Euclidean distance and migration velocity were analyzed with Imaris 9.1.2 software. Scale bars, 100 μm. Quantitative data are presented as the means ± SD (at least three independent experiments). ^∗^*P* < 0.05 and ^∗∗^*P* < 0.01. Student’s *t*-test.

Supplementary Figure 10 Depletion of LIS1 has no significant effect on the protein level of filamin A, and vice versa. A, RPE-1 cells treated with *LIS1* RNAi were subjected to western blotting analyses using anti-LIS1 and -filamin A antibodies. GAPDH, a loading control. B, RPE-1 cells treated with *filamin A* RNAi were subjected to western blotting analyses using anti-LIS1 and -filamin A antibodies. GAPDH, a loading control.

Supplementary Movie 1 The migration tracks of RPE-1 cells stably overexpressing GFP-Ctrl.

Supplementary Movie 2 The migration tracks of RPE-1 cells stably overexpressing GFP-NudC.

Supplementary Movie 3 The migration tracks of RPE-1 cells stably overexpressing GFP-NudC-L279P.
