# Corrigendum: NudC L279P mutation destabilizes filamin a by inhibiting the Hsp90 chaperoning pathway and suppresses cell migration

**DOI:** 10.3389/fcell.2023.1163790

**Published:** 2023-03-28

**Authors:** Min Liu, Zhangqi Xu, Cheng Zhang, Chunxia Yang, Jiaxing Feng, Yiqing Lu, Wen Zhang, Wenwen Chen, Xiaoyang Xu, Xiaoxia Sun, Mingyang Yang, Wei Liu, Tianhua Zhou, Yuehong Yang

**Affiliations:** ^1^ Department of Cell Biology, and Institute of Gastroenterology of the Second Affiliated Hospital, Zhejiang University School of Medicine, Hangzhou, China; ^2^ The Cancer Center of the Second Affiliated Hospital, Zhejiang University School of Medicine, Hangzhou, China; ^3^ Collaborative Innovation Center for Diagnosis and Treatment of Infectious Diseases, Hangzhou, China; ^4^ Department of Molecular Genetics, University of Toronto, Toronto, ON, Canada

**Keywords:** cell migration, filamin A, Hsp90, NudC-L279P, protein stability

In the published article, there were errors in Figure 6, Supplementary Figure S4 and the legend for Supplementary Figure S3. The representative image of the “GFP+Myc-Hsp90” group in Figure 6B and the image of the “GFP-NudC 0 h” in Supplementary Figure S4B were misused due to carelessness. In the figure legend for Supplementary Figure S3D, the description of the scale bar should be 50 μm, but not 100 μm. The corrected Figure 6, Supplementary Figure S4 and figure legend for Supplementary Figure S3 appear below.

**FIGURE 6:**
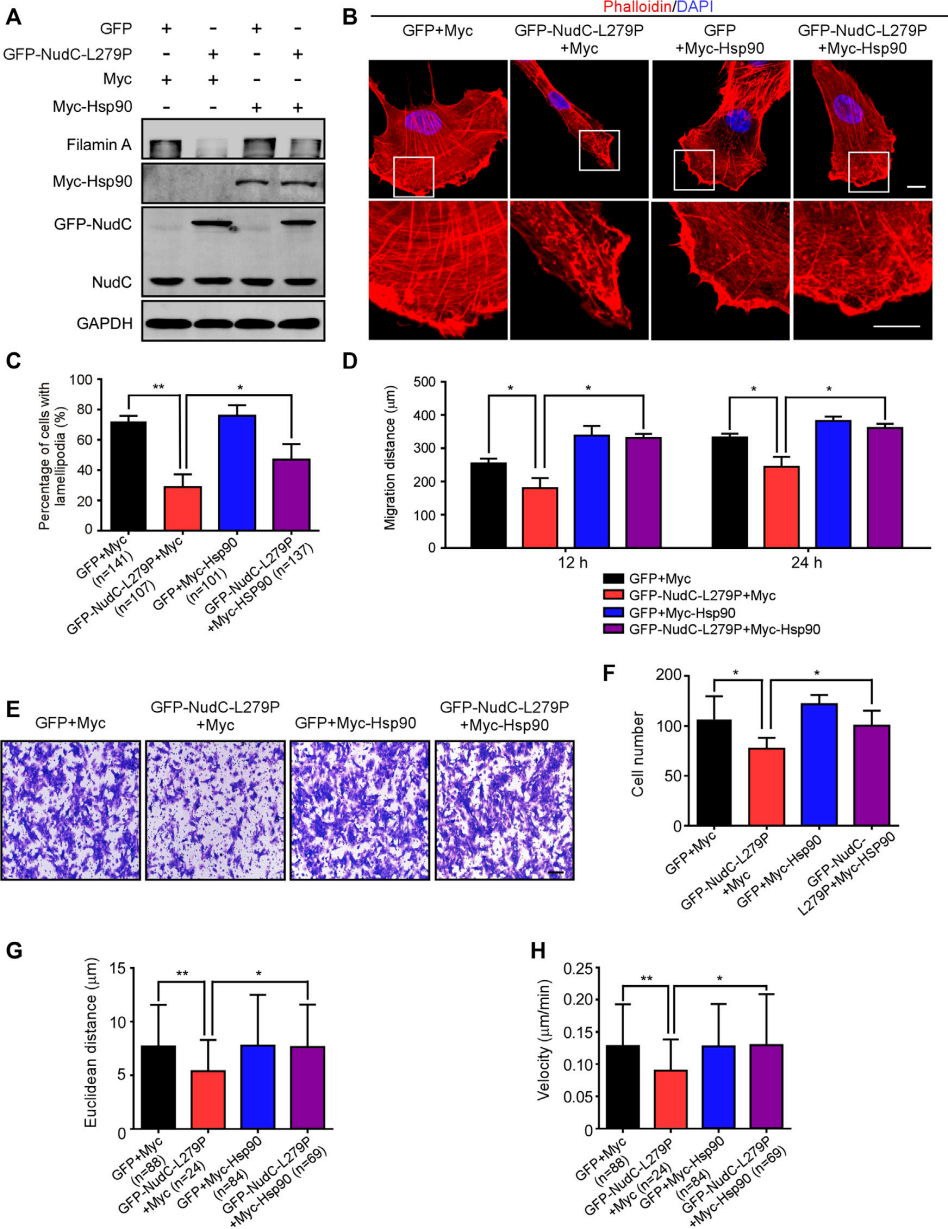
Ectopic expression of Hsp90 reverses the defects induced by NudC-L279P overexpression. RPE-1 cells stably overexpressing GFP or GFP-NudC-L279P were transfected with Myc or Myc-Hsp90 and then subjected to the following analyses. **(A)** Western blotting analysis of the expression of the indicated proteins. GAPDH, a loading control. **(B)** Cells were fixed and stained with phalloidin. DNA was visualized with DAPI. Images were captured by immunofluorescence microscopy. Scale bar, 10 μm. Higher magnifications of the boxed regions are displayed. **(C)** Cells were fixed and stained with phalloidin after 3 h of scratching. Cells with lamellipodia were counted. **(D)** Scratch wound assays detected cell motility. The distance of scratch closure was measured by ImageJ software. **(E,F)** Transwell migration assays were performed to detect cell motility. Cells that migrated to the undersides of the filters were stained with 0.2% crystal violet and monitored with DIC microscopy. The number of migrated cells per transwell was calculated. Scale bar, 100 μm. **(G,H)** The migration tracks of individual cells were traced by Imaris 9.1.2 software. Euclidean distance and migration velocity were analyzed with Imaris 9.1.2 software. Quantitative data are presented as the means ± SD (at least three independent experiments). *n*, the sample size. *p < 0.05; **p < 0.01. Student’s *t*-test.

The authors apologize for these errors and state that these mistakes do not change the scientific conclusions of the article in any way. The original article has been updated.

## Supplementary material

Supplementary Figure S3 Downregulation of filamin A suppresses cell migration in AGS cells. Cells treated with *filamin A* RNAi or not were subjected to the following analyses. **(A)** Western blotting analysis showed the expression of filamin **(A)**. GAPDH, a loading control. **(B,C)** Scratch wound assays revealed cell migration at the different time points. The scratch closure was recorded with DIC microscopy. Dashed lines indicated the approximate line of wound edges. The distance between the two edge lines was measured by ImageJ software. Scale bar, 100 μm. **(D,E)** Transwell migration assays were performed to detect cell motility. Cells that migrated to the undersides of the filters were stained with 0.2% crystal violet and monitored with DIC microscopy. The number of migrated cells per transwell was counted. Scale bar, 50 μm. **(F–H)** The migration tracks of individual cells were traced by Imaris 9.1.2 software. Euclidean distance and migration velocity were analyzed with Imaris 9.1.2 software. Quantitative data are presented as the means ± SD (at least three independent experiments). *n*, the sample size. **P* < 0.05; ***P* < 0.01. Student’s *t*-test.

Supplementary Figure S4 Overexpression of NudC-L279P suppresses cell migration in AGS Cells. AGS cells stably overexpressing GFP, GFP-NudC, or GFP-NudC-L279P were subjected to following analyses. **(A)** Western blotting analysis of the expression levels of the indicated proteins. GAPDH, a loading control. **(B,C)** Scratch wound assays revealed cell migration at the different time points. The scratch closure was recorded with fluorescence microscopy. Dashed lines defined the approximate line of wound edges. The distance between the two edge lines was measured by ImageJ software. **(D,E)** Transwell migration assays were carried out to detect cell motility. Cells that migrated to the undersides of the filters were stained with 0.2% crystal violet and monitored with DIC microscopy. The number of migrated cells per transwell was counted. **(F–H)** The migration tracks of individual cells were traced by Imaris 9.1.2 software. Euclidean distance and migration velocity were analyzed with Imaris 9.1.2 software. Scale bars, 100 μm. Quantitative data are presented as the means ± SD (at least three independent experiments). *n*, the sample size. **P* < 0.05; ***P* < 0.01; ****P* < 0.001; ns, not significant (*P* > 0.05). Student’s *t*-test.

