# Supplementary material for: NudC L279P Mutation Destabilizes Filamin A by Inhibiting the Hsp90 Chaperoning Pathway and Suppresses Cell Migration
Source: Front Cell Dev Biol. 2021 Jun 18;9:671233. doi: 10.3389/fcell.2021.671233 (PMC8273881; doi:10.3389/fcell.2021.671233)

Supplementary Figure S1

A

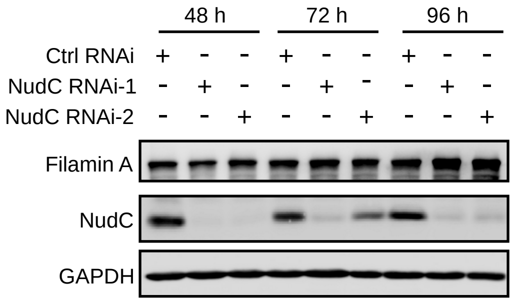

B

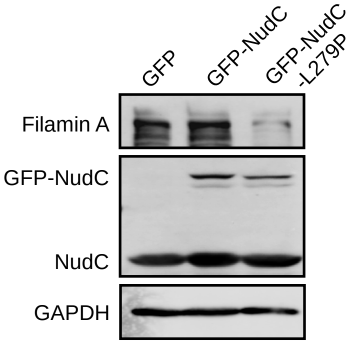

Supplementary Figure S2

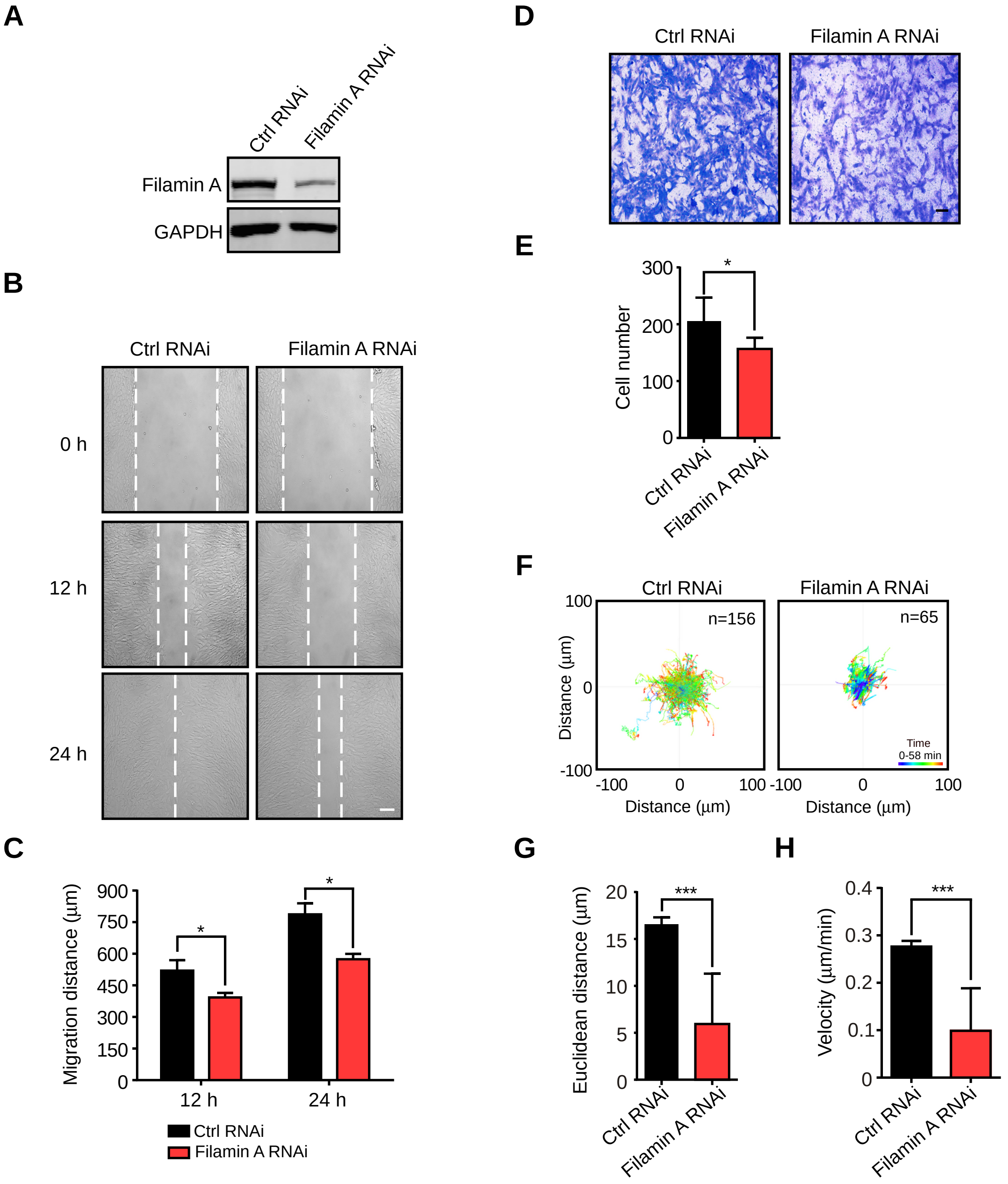

Supplementary Figure S3

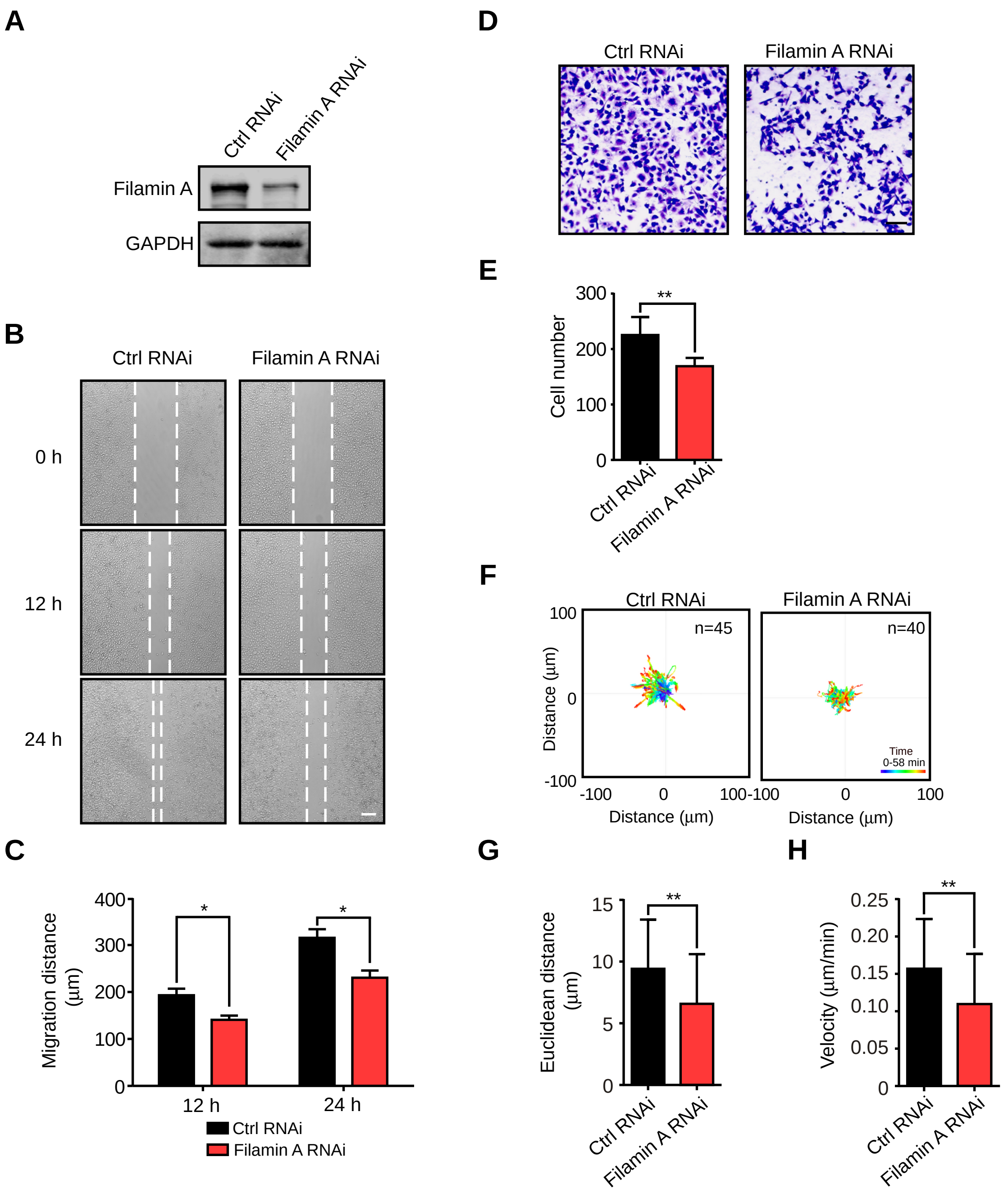

Supplementary Figure S4

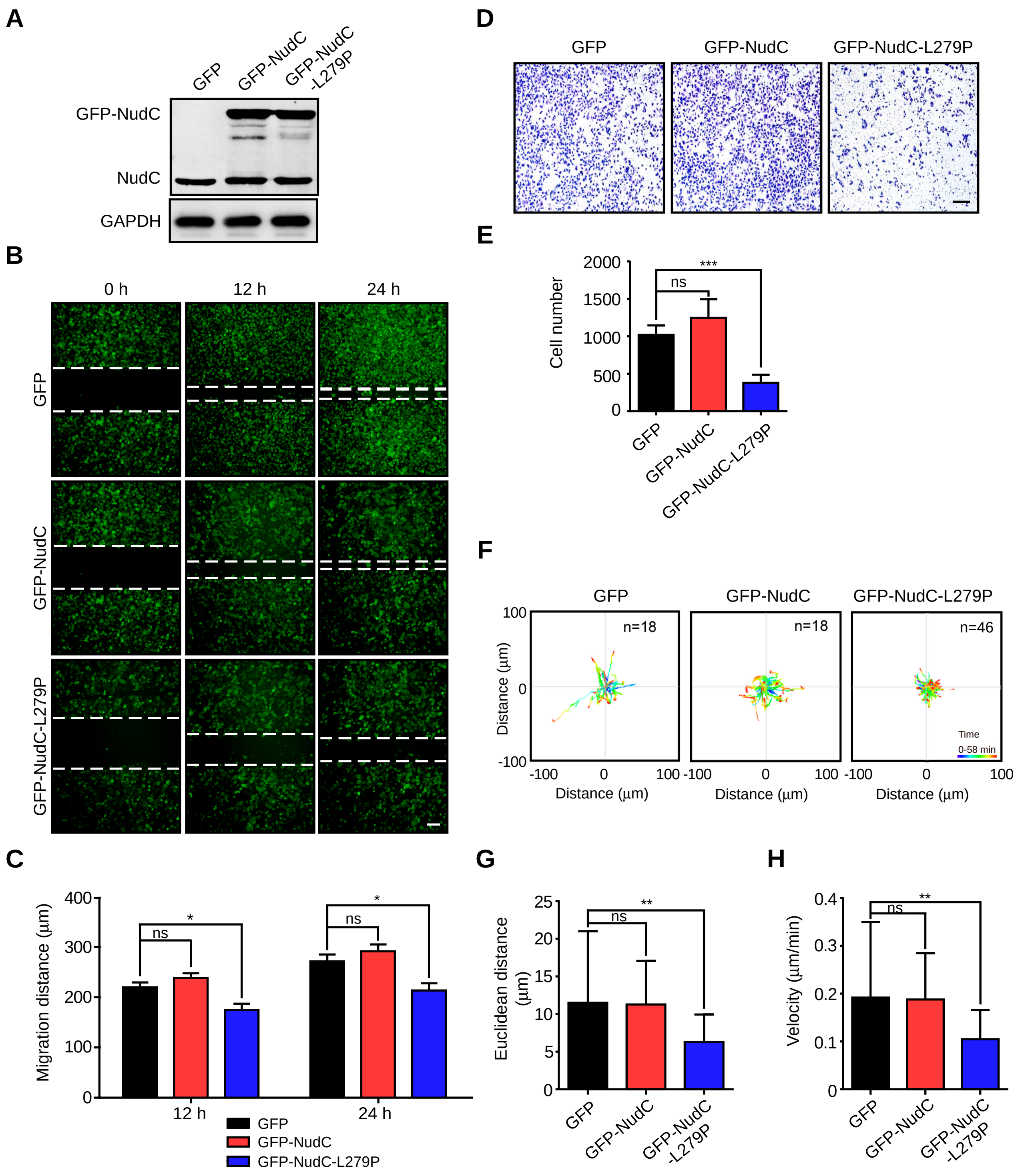

Supplementary Figure S5

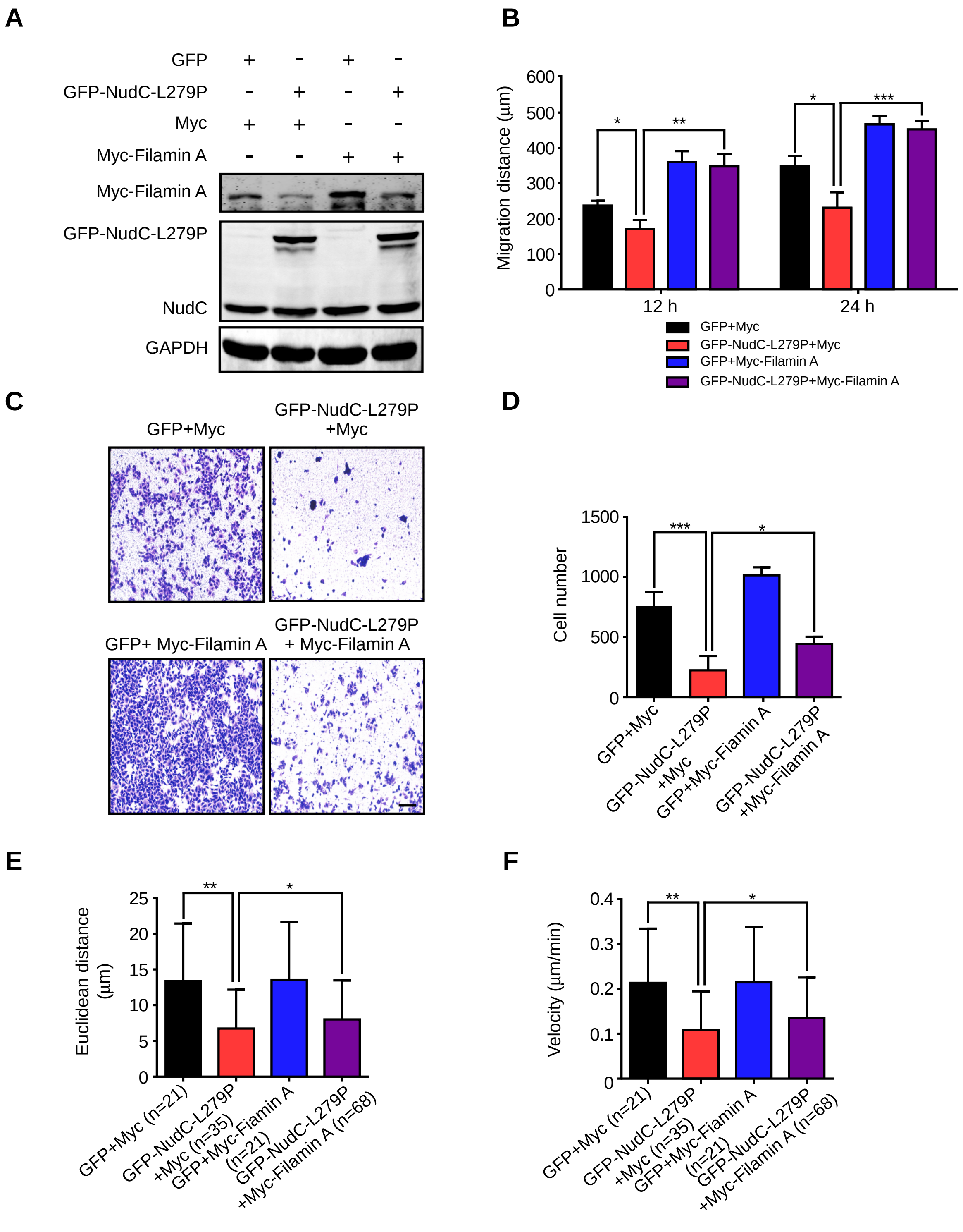

Supplementary Figure S6

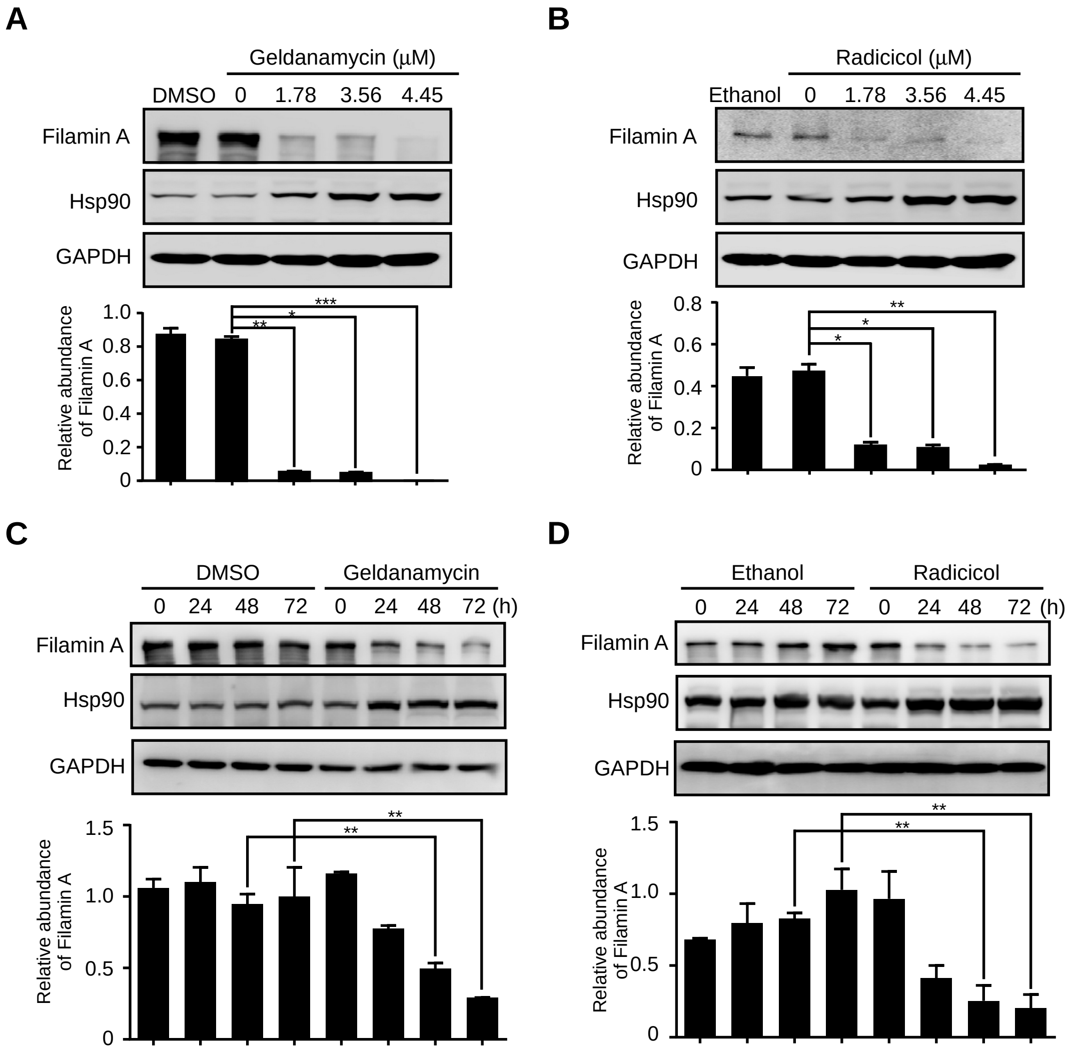

Supplementary Figure S7

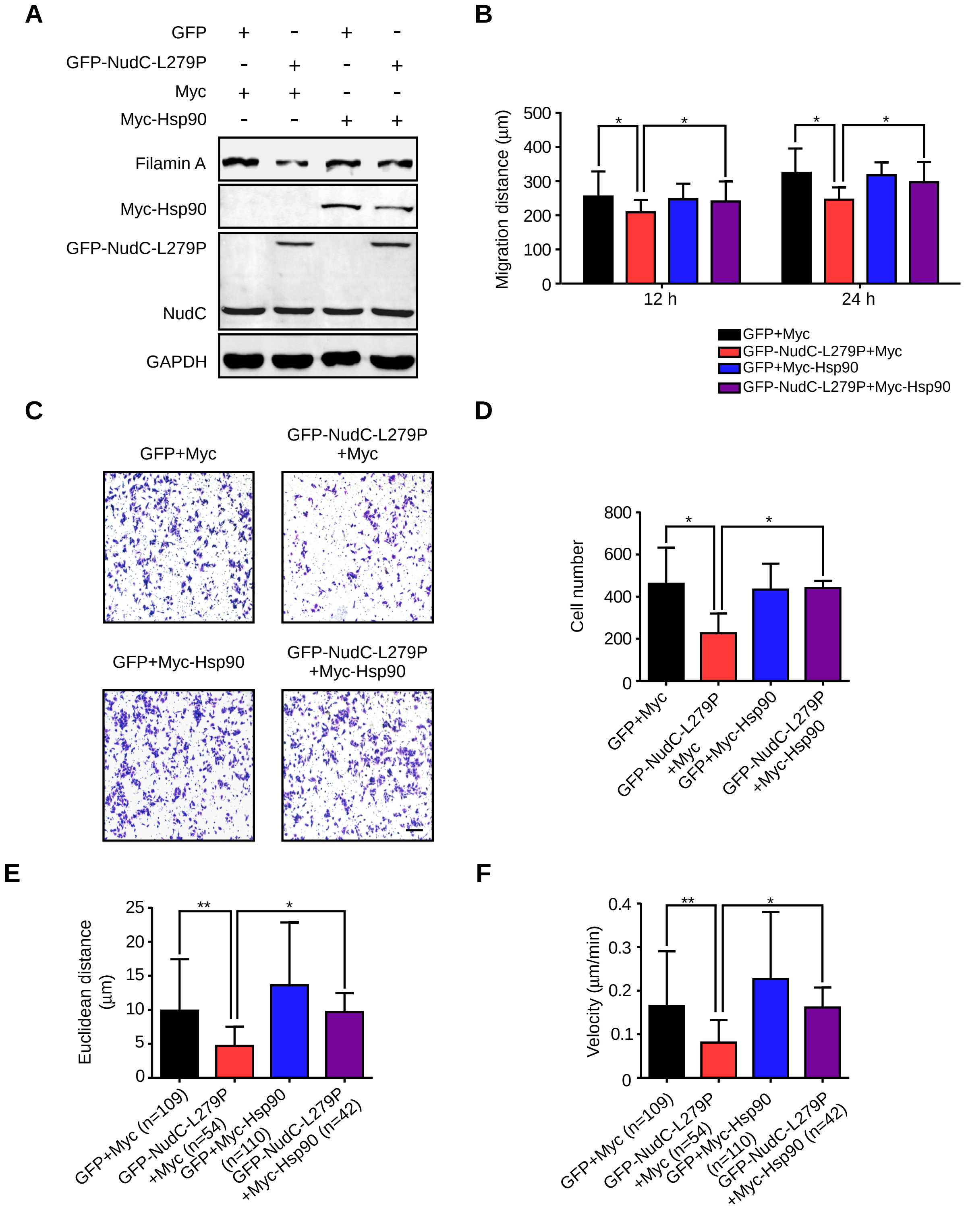

Supplementary Figure S8

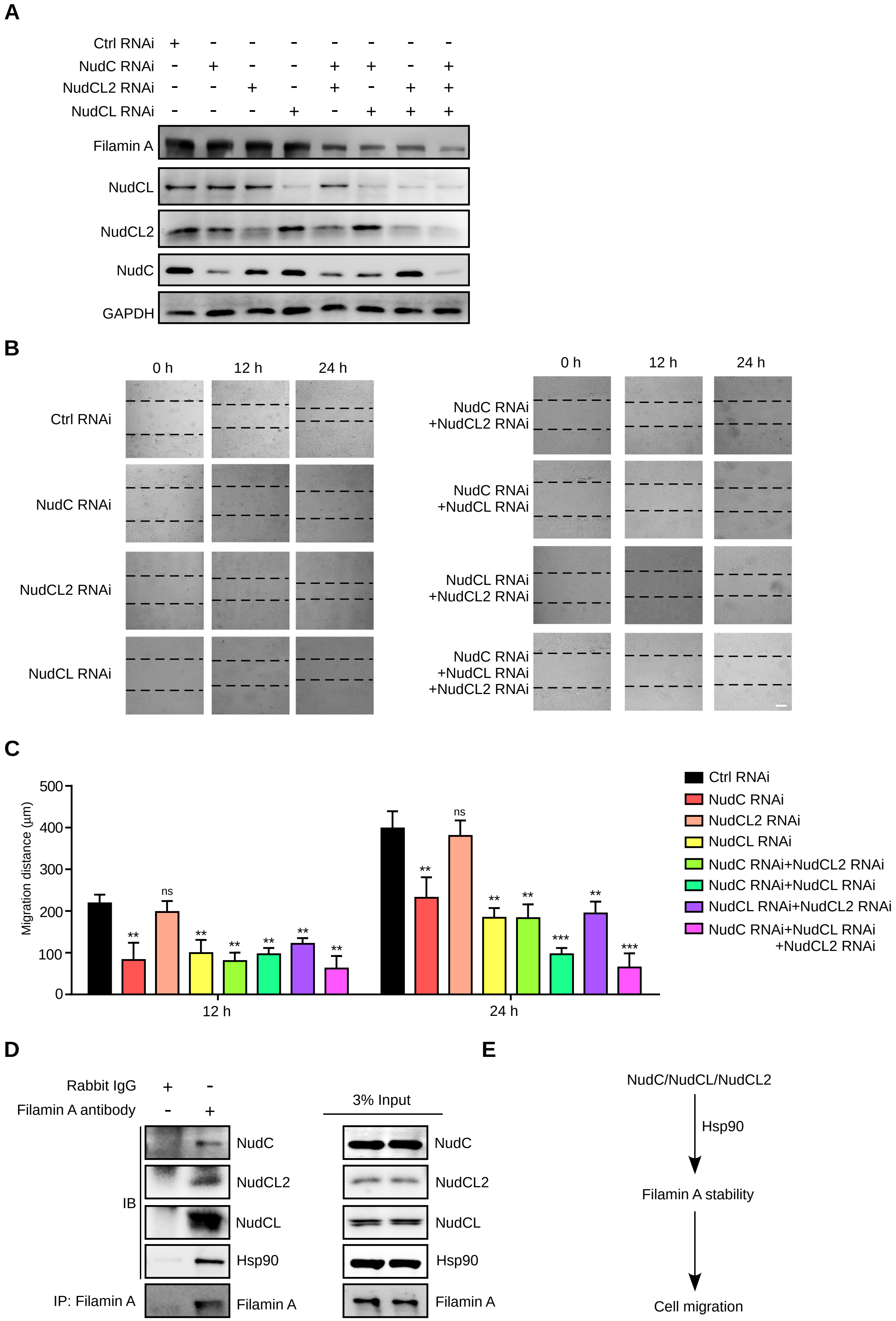

Supplementary Figure S9

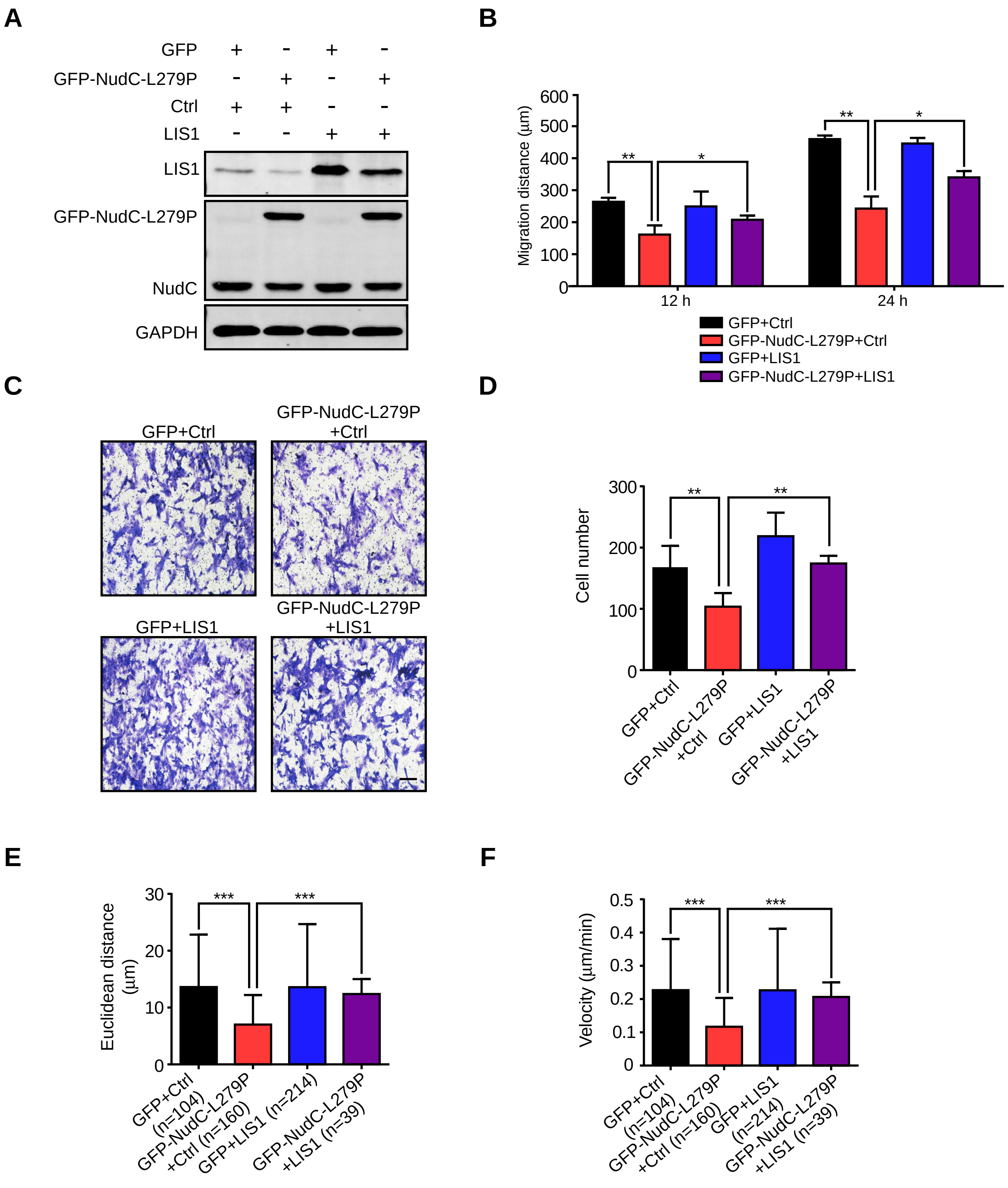

Supplementary Figure S10

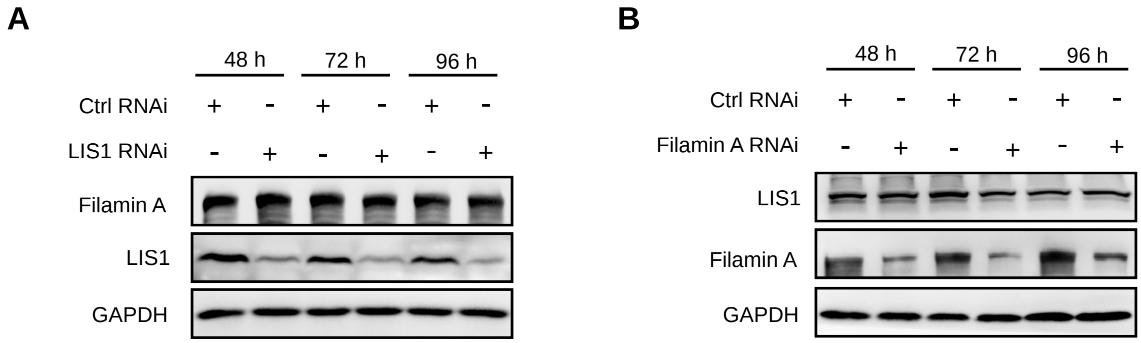

Supplement: Supplementary Figure 1 — L279P mutation of NudC destabilizes filamin A in AGS cells. (A) Cells transfected with NudC RNAi-1, -2 or control were harvested at the indicated times. The cell lysates were subjected to western blotting analysis with anti-NudC and anti-filamin A antibodies. GAPDH, a loading control. (B) Cells stably expressing GFP, GFP-NudC or GFP-NudC-L279P were subjected to western blotting analysis using the antibodies as shown. GAPDH, a loading control. [file Image_1.pdf]
