# Supplementary material for: NudC L279P Mutation Destabilizes Filamin A by Inhibiting the Hsp90 Chaperoning Pathway and Suppresses Cell Migration
Source: Front Cell Dev Biol. 2021 Jun 18;9:671233. doi: 10.3389/fcell.2021.671233 (PMC8273881; doi:10.3389/fcell.2021.671233)
Supplement: Supplementary file 5 [file Data_Sheet_1.PDF]

	UniquePepCount	Swiss-Prot accession number	gene	protein
1	30	P21333	FLNA	filamin A, alpha
2	20	O14744	PRMT5	Protein arginine N-methyltransferase 5
3	17	Q9Y2W1	THRAP3	Thyroid hormone receptor-associated protein 3
4	14	Q9BQA1	WDR77	Methylosome protein 50
5	14	P60709	ACTB	Actin, cytoplasmic 1
6	12	O43707	ACTN4	Alpha-actinin-4
7	11	P68363	TUBA1B	Tubulin alpha-1B chain
8	11	P11142	HSPA8	Isoform 1 of Heat shock cognate 71 kDa protein
9	11	P23588	EIF4B	eukaryotic translation initiation factor 4B
10	11	P68133	ACTA1	Actin, alpha skeletal muscle
11	11	P35579	MYH9	Myosin-9
12	10	Q71U36	TUBA1A	Tubulin alpha-1A chain
13	10	P02545	LMNA	Isoform A of Lamin-A/C
14	9	P68366	TUBA4A	Tubulin alpha-4A chain
15	9	P04350	TUBB	Tubulin beta chain
16	8	P08238	HSP90AB1	Heat shock protein HSP 90-beta
17	8	P11021	HSPA5	HSPA5 protein
18	8	Q15208	STK38	Serine/threonine-protein kinase 38
19	8	Q13813	SPTAN1	Splice Isoform 1 of Spectrin alpha chain, brain
20	8	P12814	ACTN1	Alpha-actinin-1
21	8	Q15149	PLEC1	Isoform 1 of Plectin-1
22	6	P07900	HSP90AA1	heat shock protein 90kDa alpha (cytosolic), class A member 1 isoform 1
23	6	P49327	FASN	Fatty acid synthase
24	6	Q00839	HNRNPU	Isoform Short of Heterogeneous nuclear ribonucleoprotein U
25	6	Q9NYF8	BCLAF1	Isoform 1 of Bcl-2-associated transcription factor 1
26	6	Q01082	SPTBN1	Isoform Long of Spectrin beta chain, brain 1
27	6	P10809	HSPD1	60 kDa heat shock protein, mitochondrial precursor
28	5	P60174	TPI1	Isoform 1 of Triosephosphate isomerase
29	5	P61978	HNRPK	Isoform 1 of Heterogeneous nuclear ribonucleoprotein K
30	5	P98175	RBM10	Putative uncharacterized protein DKFZp686E2459
31	5	P62241	RPS8	40S ribosomal protein S8
32	5	P11940	PABPC1	Isoform 1 of Polyadenylate-binding protein 1
33	5	O43795	MYO1B	Isoform 1 of Myosin-Ib
34	5	P14866	HNRNPL	heterogeneous nuclear ribonucleoprotein L isoform a
35	5	P14618	PKM2	Isoform M1 of Pyruvate kinase isozymes M1/M2
36	5	P09382	LGALS1	Galectin-1
37	5	P09651	HNRNPA1	Isoform A1-B of Heterogeneous nuclear ribonucleoprotein A1
38	5	O43390	HNRNPR	Heterogeneous nuclear ribonucleoprotein R
39	5	O75369	FLNB	Isoform 1 of Filamin-B
40	4	Q9BRS2	RIOK1	Serine/threonine-protein kinase RIO1
41	4	P36578	RPL4	60S ribosomal protein L4
42	4	P16617	PGK1	Phosphoglycerate kinase 1
43	4	P0DMV8	HSPA1A	HSPA1B Heat shock 70 kDa protein 1
44	4	Q07020	RPL18	60S ribosomal protein L18
45	4	P68104	EEF1A1	Elongation factor 1-alpha 1
46	4	P04406	GAPDH	Glyceraldehyde-3-phosphate dehydrogenase
47	4	P38159	RBMX	Heterogeneous nuclear ribonucleoprotein G
48	4	P80723	BASP1	Brain acid soluble protein 1
49	4	Q13310	PABPC4	Isoform 1 of Polyadenylate-binding protein 4
50	4	Q9Y6Y0	IVNS1ABP	Influenza virus NS1A-binding protein
51	4	P06733	ENO1	Isoform alpha-enolase of Alpha-enolase

52	4	P02786	TFRC	Transferrin receptor protein 1
53	4	Q53HC5	KLHL26	Kelch-like protein 26
54	4	O60506	SYNCRIP	Isoform 1 of Heterogeneous nuclear ribonucleoprotein Q
55	4	Q14103	HNRPD	Isoform 1 of Heterogeneous nuclear ribonucleoprotein D0
56	4	Q00610	CLTC	Isoform 1 of Clathrin heavy chain 1
57	4	Q14126	DSG2	Desmoglein-2 precursor
58	4	Q15393	SF3B3	Isoform 1 of Splicing factor 3B subunit 3
59	3	P42704	LRPPRC	Leucine-rich PPR motif-containing protein
60	3	P27274	CD59	CD59 glycoprotein precursor
61	3	P83731	RPL24	60S ribosomal protein L24
62	3	P16403	HIST1H1C	Histone H1.2
63	3	P31943	HNRPH1	Heterogeneous nuclear ribonucleoprotein H
64	3	Q99613	EIF3C	Eukaryotic translation initiation factor 3 subunit 8
65	3	P31327	CPS1	Isoform 1 of Carbamoyl-phosphate synthase [ammonia]
66	3	P22626	HNRNPA2B1	Isoform B1 of Heterogeneous nuclear ribonucleoproteins A2/B1
67	3	P00338	LDHA	Isoform 1 of L-lactate dehydrogenase A chain
68	3	P49368	CCT3	chaperonin containing TCP1, subunit 3 isoform b
69	3	P27797	CALR	Calreticulin precursor
70	3	P19338	NCL	CDNA FLJ45706 fis, clone FEBRA2028457, highly similar to Nucleolin
71	3	P17066	HSPA6	Heat shock 70 kDa protein 6
72	3	O75688	PPM1B	Isoform Beta-1 of Protein phosphatase 1B
73	3	Q9P2E9	RRBP1	Isoform 3 of Ribosome-binding protein 1
74	3	P05141	SLC25A5	ADP/ATP translocase 2
75	3	Q99729	HNRPAB	Isoform 4 of Heterogeneous nuclear ribonucleoprotein A/B
76	3	Q15365	PCBP1	Poly(rC)-binding protein 1
77	2	P62873	GNB1	Guanine nucleotide-binding protein G(I)/G(S)/G(T) subunit beta-1
78	2	P04075	ALDOA	Fructose-bisphosphate aldolase A
79	2	P62937	PPIA;PPIAL3;	Peptidyl-prolyl cis-trans isomerase A
80	2	P04792	HSPB1	Heat shock protein beta-1
81	2	P62753	RPS6	40S ribosomal protein S6
82	2	P84098	RPL19	60S ribosomal protein L19
83	2	P62899	RPL31	60S ribosomal protein L31
84	2	P11166	SLC2A1	Solute carrier family 2, facilitated glucose transporter member 1
85	2	Q09028	RBBP4	Histone-binding protein RBBP4
86	2	Q06830	PRDX1	Peroxiredoxin-1
87	2	P17844	DDX5	Probable ATP-dependent RNA helicase DDX5
88	2	P43652	ALB	Uncharacterized protein ALB
89	2	Q15942	ZYX	Zyxin
90	2	Q14157	UBAP2L	Isoform 2 of Ubiquitin-associated protein 2-like
91	2	P14625	HSP90B1	Endoplasmin precursor
92	2	Q08211	DHX9	ATP-dependent RNA helicase A
93	2	Q9Y2H1	STK38L	Serine/threonine-protein kinase 38-like
94	2	P50914	RPL14	60S ribosomal protein L14
95	2	P38646	HSPA9	Stress-70 protein, mitochondrial precursor
96	2	Q42484	RPS2	40S ribosomal protein S2
97	2	P27824	CANX	Calnexin precursor
98	2	P12277	CKB	Creatine kinase B-type
99	2	P52272	HNRPM	Isoform 1 of Heterogeneous nuclear ribonucleoprotein M
100	2	O43143	DHX15	Putative pre-mRNA-splicing factor ATP-dependent RNA helicase DHX15
101	2	P46777	RPL5	60S ribosomal protein L5
102	2	P29401	TKT	Transketolase
103	2	P14384	CPM	Carboxypeptidase M precursor

104	1	O15042	SR140	Isoform 1 of U2-associated protein SR140
105	1	P07195	LDHB	L-lactate dehydrogenase B chain
106	1	P62888	RPL30	60S ribosomal protein L30
107	1	P26373	RPL13	60S ribosomal protein L13
108	1	Q5W0B1	C13orf7	RING finger protein C13orf7
109	1	P43243	MATR3	Matrin-3
110	1	P54886	ALDH18A1	Isoform Long of Delta-1-pyrroline-5-carboxylate synthetase
111	1	Q15366	PCBP2	poly(rC)-binding protein 2 isoform b
112	1	O00159	MYO1C	Myosin-Ic
113	1	Q02878	RPL6	60S ribosomal protein L6
114	1	Q92804	TAF15	Isoform Short of TATA-binding protein-associated factor 2N
115	1	P47914	RPL29	60S ribosomal protein L29
116	1	Q9P267	MBD5	Isoform 2 of Methyl-CpG-binding domain protein 5
117	1	Q8NA68	FLJ35785	CDNA FLJ35785 fis, clone TESTI2005603, moderately similar to Homo sapiens 88-kDa Golgi protein (GM88) mRNA
118	1	P62280	RPS11	40S ribosomal protein S11
119	1	P11387	TOP1	DNA topoisomerase 1
120	1	P21291	CSRP1	Cysteine and glycine-rich protein 1
121	1	P06576	ATP5B	ATP synthase subunit beta, mitochondrial precursor
122	1	P05388	RPLP0	60S acidic ribosomal protein P0
123	1	Q14344	GNA13	Guanine nucleotide-binding protein alpha-13 subunit
124	1	Q3ZCV2	C1orf177	Isoform 2 of Uncharacterized protein C1orf177
125	1	P62906	RPL10A	60S ribosomal protein L10a
126	1	P26599	PTBP1	Isoform 1 of Polypyrimidine tract-binding protein 1
127	1	P12956	XRCC6	ATP-dependent DNA helicase 2 subunit 1
128	1	P62314	SNRPD1	Small nuclear ribonucleoprotein Sm D1
129	1	P30101	PDIA3	Protein disulfide-isomerase A3 precursor
130	1	P14678	SNRPB	Isoform SM-B' of Small nuclear ribonucleoprotein-associated proteins B and B'
131	1	Q8NC51	SERBP1	Isoform 1 of Plasminogen activator inhibitor 1 RNA-binding protein
132	1	P27694	RPA1	Replication protein A 70 kDa DNA-binding subunit
133	1	P24534	EEF1B2	Elongation factor 1-beta
134	1	Q9UHB6	LIMA1	Isoform Beta of LIM domain and actin-binding protein 1
135	1	Q13347	EIF3I	Eukaryotic translation initiation factor 3 subunit 2
136	1	O75533	SF3B1	Splicing factor 3B subunit 1
137	1	Q92901	RPL3L	60S ribosomal protein L3-like
138	1	P62266	RPS23	40S ribosomal protein S23
139	1	P07237	P4HB	Protein disulfide-isomerase precursor
140	1	P62913	RPL11	Isoform 1 of 60S ribosomal protein L11
141	1	P84103	SFRS3	Splicing factor, arginine/serine-rich 3
142	1	Q71UI9	H2AFV	Histone H2AV
143	1	Q02543	RPL18A	ribosomal protein L18a
144	1	P23396	RPS3	40S ribosomal protein S3
145	1	O95274	LYPD3	Ly6/PLAUR domain-containing protein 3 precursor
146	1	P29692	EEF1D	Elongation factor 1-delta
147	1	Q7RTV0	PHF5A	PHD finger-like domain-containing protein 5A
148	1	Q8WWY3	PRPF31	Isoform 2 of U4/U6 small nuclear ribonucleoprotein Prp31
149	1	Q14444	CAPRIN1	Caprin-1
150	1	P14923	JUP	Junction plakoglobin
151	1	Q14151	SAFB2	Scaffold attachment factor B2
152	1	P19838	NFKB1	Isoform 2 of Nuclear factor NF-kappa-B p105 subunit
153	1	Q15717	ELAVL1	ELAV-like protein 1
154	1	P51991	HNRPA3	Isoform 1 of Heterogeneous nuclear ribonucleoprotein A3
155	1	A6NMY6	ANXA2P2	Uncharacterized protein ENSP00000368744

156	1	Q12965	MYO1E	Myosin-Ie
157	1	O60812	HNRPCL1	Heterogeneous nuclear ribonucleoprotein C-like 1
158	1	Q15750	MAP3K7IP1	Mitogen-activated protein kinase kinase kinase 7-interacting protein 1
159	1	P61313	RPL15	60S ribosomal protein L15
160	1	Q14247	CTTN	Src substrate cortactin
161	1	Q13595	TRA2A	Isoform Long of Transformer-2 protein homolog
162	1	Q9UK33	ZNF580	Zinc finger protein 580
163	1	Q5GH73	XKR6	Isoform 2 of XK-related protein 6
164	1	P61247	RPS3A	40S ribosomal protein S3a
165	1	P60900	PSMA6	Proteasome subunit alpha type-6
166	1	P04843	RPN1	Dolichyl-diphosphooligosaccharide--protein glycosyltransferase 67 kDa subunit precursor
167	1	P16401	HIST1H1B	Histone H1.5
168	1	P08174	CD55	Decay-accelerating factor splicing variant 4
169	1	P00709	LALBA	Alpha-lactalbumin precursor
170	1	P13010	XRCC5	ATP-dependent DNA helicase 2 subunit 2
171	1	Q08AN1	ZNF616	Zinc finger protein 616
172	1	Q14766	LTBP1	Latent-transforming growth factor beta-binding protein, isoform 1L precursor
173	1	Q9H3R1	NDST4	Bifunctional heparan sulfate N-deacetylase/N-sulfotransferase 4
174	1	P35858	IGFALS	Insulin-like growth factor-binding protein complex acid labile chain precursor
175	1	P22314	UBE1	Ubiquitin-activating enzyme E1
176	1	Q14028	CNGB1	cyclic nucleotide gated channel beta 1
177	1	P22234	PAICS	Multifunctional protein ADE2
178	1	P53396	ACLY	ATP-citrate synthase
179	1	Q92845	KIFAP3	Kinesin-associated protein 3
180	1	Q9NTJ3	SMC4	Isoform 2 of Structural maintenance of chromosomes protein 4
181	1	P11586	MTHFD1	C-1-tetrahydrofolate synthase, cytoplasmic
182	1	O60266	ADCY3	Adenylate cyclase type 3
183	1	P53999	SUB1	Activated RNA polymerase II transcriptional coactivator p15
184	1	P08758	ANXA5	Annexin A5
185	1	P62249	RPS16	40S ribosomal protein S16
186	1	P35241	RDX	Radixin isoform b
187	1	O00567	NOL5A	Nucleolar protein 5A
188	1	O15397	IPO8	Importin-8
189	1	P35251	RFC1	Isoform 1 of Replication factor C subunit 1
190	1	O00469	PLOD2	Isoform 2 of Procollagen-lysine,2-oxoglutarate 5-dioxygenase 2 precursor
191	1	Q69YN2	CWF19L1	CWF19-like 1, cell cycle control
192	1	Q9NW15	TMEM16K	Isoform 1 of Transmembrane protein 16K
193	1	Q96BJ3	C1orf80	Isoform 1 of UPF0491 protein C1orf80
194	1	Q5SRN2	C6orf10	Testis specific basic protein
195	1	Q9NUN8	FLJ11235	Putative uncharacterized protein FLJ11235
196	1	I7GEV6	FLJ41327	CDNA FLJ41327 fis, clone BRAMY2047169
197	1	Q96B86	RGMA	Isoform 2 of Repulsive guidance molecule A precursor
198	1	P67809	YBX1	Nuclease sensitive element-binding protein 1
199	1	P62424	RPL7A	60S ribosomal protein L7a
200	1	Q96QZ7	MAGI1	Isoform 2 of Membrane-associated guanylate kinase, WW and PDZ domain-containing protein 1
201	1	Q14008	CKAP5	Cytoskeleton-associated protein 5
202	1	P35659	DEK	Protein DEK
203	1	Q9Y6G9	DYNC1LI1	Cytoplasmic dynein 1 light intermediate chain 1
204	1	P31689	DNAJA1	DnaJ homolog subfamily A member 1
205	1	Q9Y2K9	STXBP5L	Isoform 1 of Syntaxin-binding protein 5-like

206	1	O95382	MAP3K6	Isoform 1 of Mitogen-activated protein kinase kinase kinase 6
207	1	P13640	MT1G	Isoform 1 of Metallothionein-1G
208	1	Q03405	PLAUR	Isoform 1 of Urokinase plasminogen activator surface receptor precursor
209	1	Q13151	HNRNPA0	Heterogeneous nuclear ribonucleoprotein A0
210	1	Q12797	ASPH	aspartate beta-hydroxylase isoform c
211	1	P13639	EEF2	Elongation factor 2
212	1	Q5VT06	CEP350	Centrosome-associated protein 350
213	1	P06744	GPI	Glucose-6-phosphate isomerase
214	1	Q9P219	CCDC88C	similar to DVL-binding protein DAPLE isoform 2
215	1	O94979	SEC31A	Isoform 3 of Protein transport protein Sec31A
216	1	Q12829	RAB40B	Ras-related protein Rab-40B
217	1	P83369	LSM11	U7 snRNA-associated Sm-like protein LSm11
218	1	Q9GZV4	EIF5A2	Eukaryotic translation initiation factor 5A-2
219	1	Q8WUA7	TBC1D22A	Isoform 1 of TBC1 domain family member 22A
220	1	P61160	ACTR2	Actin-related protein 2
221	1	P55209	NAP1L1	Nucleosome assembly protein 1-like 1
222	1	Q96SI9	STRBP	Isoform 1 of Spermatid perinuclear RNA-binding protein
223	1	O75494	SFRS10	Splicing factor, arginine/serine-rich 10
224	1	Q12872	SFRS8	Splicing factor, arginine/serine-rich 8
225	1	P45880	VDAC2	Voltage-dependent anion-selective channel protein 2
226	1	P57088	TMEM33	Transmembrane protein 33
227	1	P51114	FXR1	Isoform 1 of Fragile X mental retardation syndrome-related protein 1
228	1	P43115	PTGER3	Isoform EP3E of Prostaglandin E2 receptor EP3 subtype
229	1	P55884	EIF3B	Isoform 1 of Eukaryotic translation initiation factor 3 subunit 9
230	1	P30050	RPL12	60S ribosomal protein L12
231	1	Q14498	RBM39	Isoform 1 of RNA-binding protein 39
232	1	P78527	PRKDC	Isoform 1 of DNA-dependent protein kinase catalytic subunit
233	1	Q63HR2	TENC1	Isoform 2 of Tensin-like C1 domain-containing phosphatase
234	1	O75159	SOCS5	Suppressor of cytokine signaling 5
235	1	P35232	PHB	Prohibitin
236	1	Q08945	SSRP1	FACT complex subunit SSRP1
237	1	P23246	SFPQ	Isoform Long of Splicing factor, proline- and glutamine-rich
238	1	Q96JQ0	DCHS1	Protocadherin-16 precursor
239	1	P31153	MAT2A	S-adenosylmethionine synthetase isoform type-2
240	1	P34932	HSPA4	Heat shock 70 kDa protein 4
241	1	O94813	SLIT2	Slit homolog 3 protein precursor
242	1	Q9NR30	DDX21	Isoform 1 of Nucleolar RNA helicase 2
243	1	P13674	P4HA1	Isoform 1 of Prolyl 4-hydroxylase subunit alpha-1 precursor
244	1	P21589	NT5E	5'-nucleotidase precursor
245	1	P46087	NOL1	94 kDa protein
246	1	O95831	AIFM1	Isoform 1 of Apoptosis-inducing factor 1, mitochondrial precursor
247	1	Q96SB3	PPP1R9B	Conserved hypothetical protein
248	1	P18124	RPL7	60S ribosomal protein L7
249	1	Q13242	SFRS9	Splicing factor, arginine/serine-rich 9
250	1	Q9HAU5	UPF2	CDNA FLJ23795 fis, clone HEP22107
251	1	P17096	HMGA1	Isoform HMG-I of High mobility group protein HMG-I/HMG-Y
252	1	O14598	VCY	VCY1B Testis-specific basic protein Y 1
253	1	P08670	VIM	Vimentin
254	1	P14550	AKR1A1	Alcohol dehydrogenase
255	1	Q9P2J3	KLHL9	Kelch-like protein 9
256	1	Q14764	MVP	Major vault protein
257	1	P31948	STIP1	Stress-induced-phosphoprotein 1

258	1	Q6UWQ7	IGFL2	LOC401923 CDNA FLJ43766 fis, clone TESTI2049246
259	1	O60832	DKC1	H/ACA ribonucleoprotein complex subunit 4
260	1	P02100	HBE1	Hemoglobin subunit epsilon
261	1	Q9UM47	NOTCH3	Neurogenic locus notch homolog protein 3 precursor
262	1	Q92608	DOCK2	Isoform 1 of Dedicator of cytokinesis protein 2
263	1	Q14781	CBX2	Isoform 1 of Chromobox protein homolog 2
264	1	P24752	ACAT1	Acetyl-CoA acetyltransferase, mitochondrial precursor
265	1	P05023	ATP1A1	Isoform Long of Sodium/potassium-transporting ATPase subunit alpha-1 precursor
266	1	Q5DX21	IGSF11	CDNA FLJ40348 fis, clone TESTI2033300
267	1	P62854	RPS26	40S ribosomal protein S26
268	1	Q01081	U2AF1	Splicing factor U2AF 35 kDa subunit
269	1	Q15427	SF3B4	Splicing factor 3B subunit 4
270	1	Q96HS1	PGAM5	Isoform 2 of Phosphoglycerate mutase family member 5 precursor
271	1	P0C1Z6	TFPT	22 kDa protein
272	1	Q9H252	KCNH6	Isoform 1 of Potassium voltage-gated channel subfamily H member 6
273	1	P22105	TNXB	Isoform XB of Tenascin-X precursor
274	1	Q96I13	ABHD8	Abhydrolase domain-containing protein 8
275	1	Q01851	POU4F1	POU domain, class 4, transcription factor 1
276	1	P35052	GPC1	Glypican-1 precursor
277	1	Q9HCE3	ZNF532	Zinc finger protein 532
278	1	P26640	VARS	Valyl-tRNA synthetase
279	1	O94875	SORBS2	55 kDa protein
280	1	Q6WCQ1	M-RIP	myosin phosphatase-Rho interacting protein isoform 1
281	1	P31942	HNRPH3	Isoform 1 of Heterogeneous nuclear ribonucleoprotein H3
282	1	P57789	KCNK10	Isoform A of Potassium channel subfamily K member 10
283	1	P25705	ATP5A1	ATP synthase subunit alpha, mitochondrial precursor
284	1	Q9UQ35	SRRM2	Isoform 1 of Serine/arginine repetitive matrix protein 2
285	1	P50402	EMD	Emerin
286	1	Q8TDJ6	DMXL2	DmX-like protein 2
287	1	P09874	PARP1	Poly [ADP-ribose] polymerase 1
288	1	P46778	RPL21	60S ribosomal protein L21
289	1	P49792	RANBP2	E3 SUMO-protein ligase RanBP2
290	1	P55265	ADAR	Isoform 2 of Double-stranded RNA-specific adenosine deaminase
