# Supplementary material for: NudC L279P Mutation Destabilizes Filamin A by Inhibiting the Hsp90 Chaperoning Pathway and Suppresses Cell Migration
Source: Front Cell Dev Biol. 2021 Jun 18;9:671233. doi: 10.3389/fcell.2021.671233 (PMC8273881; doi:10.3389/fcell.2021.671233)
Supplement: Supplementary file 6 [file Data_Sheet_2.PDF]

	UniquePep Count	Swiss-Prot accession number	gene	protein
1	21	P21333	FLNA	Filamin A, alpha
2	18	O14744	PRMT5	Protein arginine N-methyltransferase 5
3	17	Q9Y608	LRRFIP2	Leucine-rich repeat flightless-interacting protein 2
4	12	P10809	HSPD1	60 kDa heat shock protein, mitochondrial precursor
5	11	P61978-1	HNRNPK	Splice Isoform 1 of Heterogeneous nuclear ribonucleoprotein K
6	9	O75688-1	PPM1B	Serine/threonine-protein kinase 38
7	8	P68363	TUBA1B	Tubulin alpha-1B chain
8	8	P30050	RPL12	60S ribosomal protein L12
9	7	P60709	ACTB	Actin, cytoplasmic 1
10	7	P35579	MYH9	Myosin-9
11	6	P11142-1	HSPA8	Heat shock cognate 71 kDa protein
12	6	Q9Y2W1	THRAP3	Thyroid hormone receptor-associated protein 3
13	6	P23588	EIF4B	Eukaryotic translation initiation factor 4B
14	5	P52597	HNRNPF	Heterogeneous nuclear ribonucleoprotein F
15	5	Q9Y6Y0	IVNS1ABP	Influenza virus NS1A-binding protein
16	5	A6NHL2	TUBAL3	Tubulin alpha chain-like 3
17	4	P07437	TUBB	Tubulin beta chain
18	4	Q14764	MVP	Major vault protein
19	4	Q9NYF8-1	BCLAF1	Splice Isoform 1 of Bcl-2-associated transcription factor 1
20	3	P09651-1	HNRNPA1	heterogeneous nuclear ribonucleoprotein A1 isoform b
21	3	P68104	EEF1A1	Elongation factor 1-alpha 1
22	3	O60506-1	SYNCRIP	Splice Isoform 1 of Heterogeneous nuclear ribonucleoprotein Q
23	3	Q01082-1	SPTBN1	Splice Isoform Long of Spectrin beta chain, brain 1
24	3	P62314	SNRPD1	Small nuclear ribonucleoprotein Sm D1
25	3	P54886-1	ALDH18A1	Splice Isoform Long of Delta 1-pyrroline-5-carboxylate synthetase
26	3	Q13813-1	SPTAN1	Splice Isoform 1 of Spectrin alpha chain, brain
27	3	A6NMY6	ANXA2P2	Putative annexin A2-like protein
28	3	Q18PE1	DOK7	Protein Dok-7
29	3	P07900	HSP90AA1	Heat shock protein HSP 90-alpha
30	3	P67936-1	TPM4	Tropomyosin 4
31	3	P62258	YWHAE	14-3-3 protein epsilon
32	3	P05388	RPLP0	60S acidic ribosomal protein P0
33	3	P81605	DCD	Dermcidin precursor
34	2	B9ZVP1	HNRNPA2B1	Putative uncharacterized protein HNRNPA2B1
35	2	P05387	RPLP2	60S acidic ribosomal protein P2
36	2	P48643	CCT5	T-complex protein 1 subunit epsilon
37	2	P62424	RPL7A	60S ribosomal protein L7a
38	2	P98175	RBM10	RNA binding motif protein 10 isoform 2
39	2	Q02878	RPL6	60S ribosomal protein L6
40	2	P68371	TUBB2C	Tubulin beta-2C chain
41	2	P14866	HNRNPL	heterogeneous nuclear ribonucleoprotein L isoform a
42	2	P14618-2	PKM2	Pyruvate kinase isozymes M1/M2
43	2	P54105	CLNS1A	Methylosome subunit pICln
44	2	P06748-2	NPM1	Splice Isoform 2 of Nucleophosmin
45	2	P04406	GAPDH	Glyceraldehyde-3-phosphate dehydrogenase
46	2	P55072	VCP	Transitional endoplasmic reticulum ATPase
47	2	Q8NCM8	DYNC2H1	Cytoplasmic dynein 2 heavy chain 1
48	2	P07737	PFN1	Profilin-1
49	2	Q16795	NDUFA9	NADH dehydrogenase [ubiquinone] 1 alpha subcomplex subunit 9
50	2	P84090	ERH	Enhancer of rudimentary homolog
51	2	P05386	RPLP1	60S acidic ribosomal protein P1
52	2	P02768-1	ALB	Serum albumin
53	2	Q07020	RPL18	60S ribosomal protein L18

54	2	P62241	RPS8	40S ribosomal protein S8
55	1	P00338-1	LDHB	lactate dehydrogenase A
56	1	P09104	ENO2	Gamma-enolase
57	1	Q8IWY7	TTBK2	Tau-tubulin kinase
58	1	P42704	LRPPRC	Leucine-rich PPR motif-containing protein, mitochondrial precursor
59	1	Q15366	PCBP2	poly(rC)-binding protein 2
60	1	P67809	YBX1	Nuclease sensitive element-binding protein 1
61	1	P05161	ISG15	Interferon-induced 17 kDa protein precursor
62	1	Q07021	C1QBP	Complement component 1, Q subcomponent-binding protein
63	1	P42166	TMPO	Lamina-associated polypeptide 2 isoform alpha
64	1	P36578	RPL4	60S ribosomal protein L4
65	1	Q6ZS99		CDNA FLJ45706 fis, clone FEBRA2028457, highly similar to Nucleolin
66	1	Q5SZL2	C6orf204	Coiled-coil domain-containing protein C6orf204
67	1	P62280	RPS11	40S ribosomal protein S11
68	1	P05141	SLC25A5	ADP/ATP translocase 2
69	1	Q9GZU2-1	PEG3	Paternally expressed gene 3 isoform 1
70	1	P09382	LGALS1	Galectin-1
71	1	P17987	CCT1	T-complex protein 1 subunit alpha
72	1	Q8NB14	USP38	Ubiquitin carboxyl-terminal hydrolase 38
73	1	Q8WYA0-3	IFT81	Intraflagellar transport protein 81 homolog
74	1	Q05682-1	CALD1	Splice Isoform 1 of Caldesmon
75	1	P04075	ALDOA	Fructose-bisphosphate aldolase A
76	1	P62277	RPS13	40S ribosomal protein S13
77	1	P14678-1	SNRPB	Splice Isoform SM-B' of Small nuclear ribonucleoprotein associated proteins B and B'
78	1	P62269	RPS18	40S ribosomal protein S18
79	1	P80723	BASP1	Brain acid soluble protein 1
80	1	Q96E39	RBMXL1	RNA binding motif protein, X-linked-like 1
81	1	Q13151	HNRNPA0	Heterogeneous nuclear ribonucleoprotein A0
82	1	P05109	S100A8	Protein S100-A8
83	1	P62851	RPS25	40S ribosomal protein S25
84	1	Q99832	CCT7	T-complex protein 1 subunit eta (a subunit of chaperonin CCT)
85	1	P50914	RPL14	60S ribosomal protein L14
86	1	P62888	RPL30	60S ribosomal protein L30
87	1	Q9UBB4	ATXN10	Ataxin-10
88	1	P62910	RPL32	60S ribosomal protein L32
89	1	Q96DG6	CMBL	Carboxymethylenebutenolidase homolog
90	1	Q9Y2E6	DTX4	deltex 4 homolog
91	1	Q9P287-1	BCCIP	BRCA2 and CDKN1A-interacting protein, isoform BCCIPbeta
92	1	P46781	RPS9	40S ribosomal protein S9
93	1	Q13442	PDAP1	28 kDa heat- and acid-stable phosphoprotein
94	1	P50452	SERPINB8	Serpin B8
95	1	P46782	RPS5	40S ribosomal protein S5
96	1	P62263	RPS14	40S ribosomal protein S14
97	1	P39023	RPL3	similar to ribosomal protein L3
98	1	P11177-1	PDHB	Splice Isoform 1 of Pyruvate dehydrogenase E1 component beta subunit
99	1	Q13045	FLII	Protein flightless-1 homolog
100	1	Q99873	PPMT1	Splice Isoform 2 of Protein arginine N-methyltransferase 1
101	1	P62701	RPS4X	40S ribosomal protein S4, X isoform
102	1	P55036-1	PSMD4	Splice Isoform Rpn10A of 26S proteasome non-ATPase regulatory subunit 4
103	1	Q14444	CAPRIN1	Cytoplasmic activation- and proliferation-associated protein 1
104	1	Q02413	DSG1	Desmoglein-1 precursor
105	1	A6NL28		Putative tropomyosin alpha-3 chain-like protein
106	1	P62917	RPL8	60S ribosomal protein L8
107	1	Q15057	ACAP2	rf-GAP with coiled-coil, ANK repeat and PH domain-containing protein 2

108	1	O14924-1	RGS12	Splice Isoform 1 of Regulator of G-protein signaling 12
109	1	P62857	RPS28	40S ribosomal protein S28
110	1	P12277	CKB	Creatine kinase B-type
111	1	Q15393	SF3B3	Splicing factor 3B subunit 3
112	1	P06310		Ig kappa chain V-II region RPMI 6410 precursor
113	1	Q9NXV2	KCTD5	BTB/POZ domain-containing protein KCTD5
114	1	Q9GZT3	C14orf156	SRA stem-loop-interacting RNA-binding protein, mitochondrial
115	1	P29692	EEF1D	Elongation factor 1-delta
116	1	P26641	EEF1G	Elongation factor 1-gamma
117	1	P62805	HIST4H4	Histone H4
118	1	P17096-1	HMGA1	high mobility group AT-hook 1 isoform a
119	1	O14818	PSMA7	Splice Isoform 1 of Proteasome subunit alpha type 7
120	1	Q96NA8	TSNARE1	t-SNARE domain-containing protein 1
121	1	P25398	RPS12	60S ribosomal protein L12
122	1	P38646	HSPA9	Stress-70 protein, mitochondrial precursor
123	1	P37802	TAGLN2	Transgelin-2
124	1	Q99759	MAP3K3	mitogen-activated protein kinase kinase kinase 3 isoform 2
125	1	Q8NE31	FAM13C	hypothetical protein LOC220965 isoform 2
126	1	P13639	EEF2	Elongation factor 2
127	1	P12814	ACTN1	Alpha-actinin-1
128	1	P62318	SNRPD3	Small nuclear ribonucleoprotein Sm D3
129	1	Q86W42	THOC6	THO complex subunit 6 homolog
130	1	P61247	RPS3A	40S ribosomal protein S3a
131	1	P42766	RPL35	similar to 60S ribosomal protein L35
