# Supplementary material for: NudC L279P Mutation Destabilizes Filamin A by Inhibiting the Hsp90 Chaperoning Pathway and Suppresses Cell Migration
Source: Front Cell Dev Biol. 2021 Jun 18;9:671233. doi: 10.3389/fcell.2021.671233 (PMC8273881; doi:10.3389/fcell.2021.671233)
Supplement: Supplementary file 7 [file Data_Sheet_3.PDF]

	Swiss-Prot	gene	protein
1	P21333	FLNA	filamin A, alpha
2	O14744	PRMT5	Protein arginine N-methyltransferase 5
3	Q9Y2W1	THRAP3	Thyroid hormone receptor-associated protein 3
4	P60709	ACTB	Actin, cytoplasmic 1
5	P68363	TUBA1B	Tubulin alpha-1B chain
6	P11142	HSPA8	Isoform 1 of Heat shock cognate 71 kDa protein
7	P23588	EIF4B	eukaryotic translation initiation factor 4B
8	P35579	MYH9	Myosin-9
9	P04350	TUBB	Tubulin beta chain
10	Q13813	SPTAN1	Splice Isoform 1 of Spectrin alpha chain, brain
11	P12814	ACTN1	Alpha-actinin-1
12	P07900	HSP90AA1	Heat shock protein HSP 90-alpha
13	Q9NYF8-1	BCLAF1	Splice Isoform 1 of Bcl-2-associated transcription factor 1
14	Q01082-1	SPTBN1	Splice Isoform Long of Spectrin beta chain, brain 1
15	P10809	HSPD1	60 kDa heat shock protein, mitochondrial precursor
16	P98175	RBM10	RNA binding motif protein 10 isoform 2
17	P62241	RPS8	40S ribosomal protein S8
18	P14866	HNRNPL	heterogeneous nuclear ribonucleoprotein L isoform a
19	P14618-2	PKM2	Pyruvate kinase isozymes M1/M2
20	P09382	LGALS1	Galectin-1
21	P09651-1	HNRNPA1	heterogeneous nuclear ribonucleoprotein A1 isoform b
22	P36578	RPL4	60S ribosomal protein L4
23	Q07020	RPL18	60S ribosomal protein L18
24	P68104	EEF1A1	Elongation factor 1-alpha 1
25	P04406	GAPDH	Glyceraldehyde-3-phosphate dehydrogenase
26	P80723	BASP1	Brain acid soluble protein 1
27	Q9Y6Y0	IVNS1ABP	Influenza virus NS1A-binding protein
28	O60506-1	SYNCRIP	Splice Isoform 1 of Heterogeneous nuclear ribonucleoprotein Q
29	Q15393	SF3B3	Splicing factor 3B subunit 3
30	P42704	LRPPRC	Leucine-rich PPR motif-containing protein, mitochondrial precursor
31	B9ZVP1	HNRNPA2B1	Putative uncharacterized protein HNRNPA2B1
32	O75688-1	PPM1B	Protein phosphatase 1B
33	P05141	SLC25A5	ADP/ATP translocase 2
34	P04075	ALDOA	Fructose-bisphosphate aldolase A
35	P02768-1	ALB	Serum albumin
36	P50914	RPL14	60S ribosomal protein L14
37	P38646	HSPA9	Stress-70 protein, mitochondrial precursor
38	P12277	CKB	Creatine kinase B-type
39	P00338-1	LDHB	lactate dehydrogenase A
40	P62888	RPL30	60S ribosomal protein L30
41	P54886-1	ALDH18A1	Splice Isoform Long of Delta 1-pyrroline-5-carboxylate synthetase
42	Q15366	PCBP2	poly(rC)-binding protein 2
43	Q02878	RPL6	60S ribosomal protein L6
44	P62280	RPS11	40S ribosomal protein S11
45	P05388	RPLP0	60S acidic ribosomal protein P0
46	P62314	SNRPD1	Small nuclear ribonucleoprotein Sm D1
47	P14678-1	SNRPB	Splice Isoform SM-B' of Small nuclear ribonucleoprotein associated pro
48	P29692	EEF1D	Elongation factor 1-delta
49	Q14444	CAPRIN1	Cytoplasmic activation- and proliferation-associated protein 1
50	A6NMY6	ANXA2P2	Putative annexin A2-like protein
51	P61247	RPS3A	40S ribosomal protein S3a
52	P67809	YBX1	Nuclease sensitive element-binding protein 1
53	P62424	RPL7A	60S ribosomal protein L7a

54	Q13151	HNRNPA0	Heterogeneous nuclear ribonucleoprotein A0
55	P13639	EEF2	Elongation factor 2
56	P17096-1	HMGA1	high mobility group AT-hook 1 isoform a
57	Q14764	MVP	Major vault protein
58	P30050	RPL12	60S ribosomal protein L12

teins B and
