# Supplementary material for: Corrigendum: NudC L279P mutation destabilizes filamin a by inhibiting the Hsp90 chaperoning pathway and suppresses cell migration
Source: Front Cell Dev Biol. 2023 Mar 28;11:1163790. doi: 10.3389/fcell.2023.1163790 (PMC10086320; doi:10.3389/fcell.2023.1163790)

Supplementary Figure S1

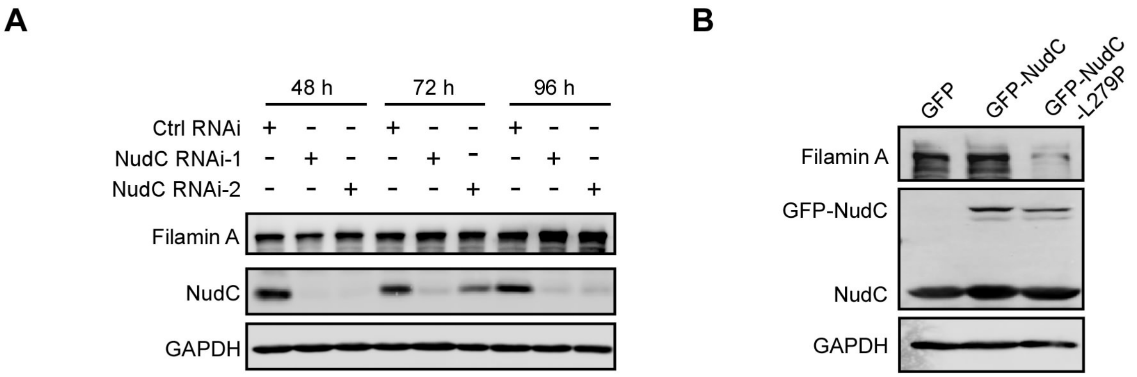

Supplementary Figure S2

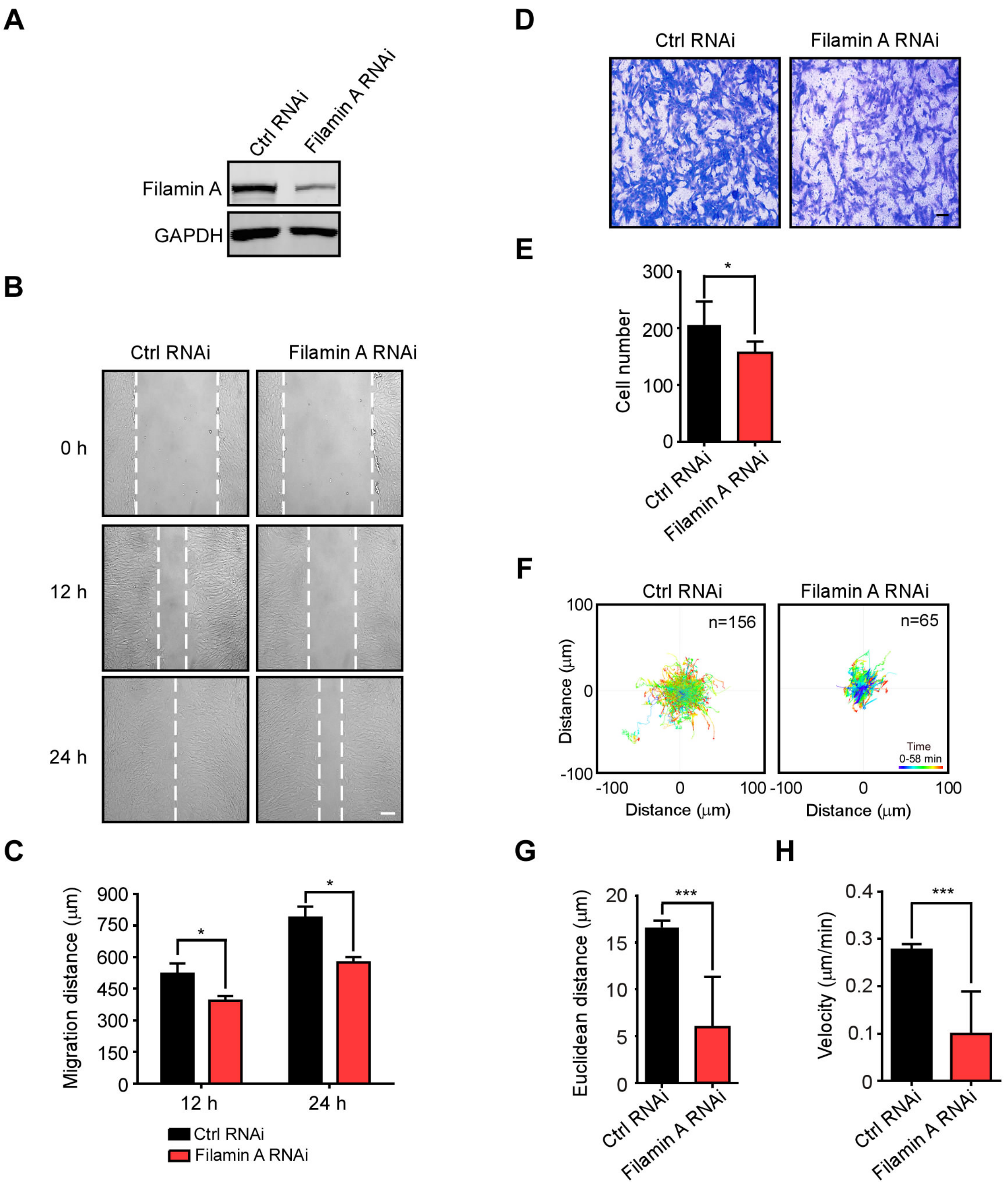

Supplementary Figure S3

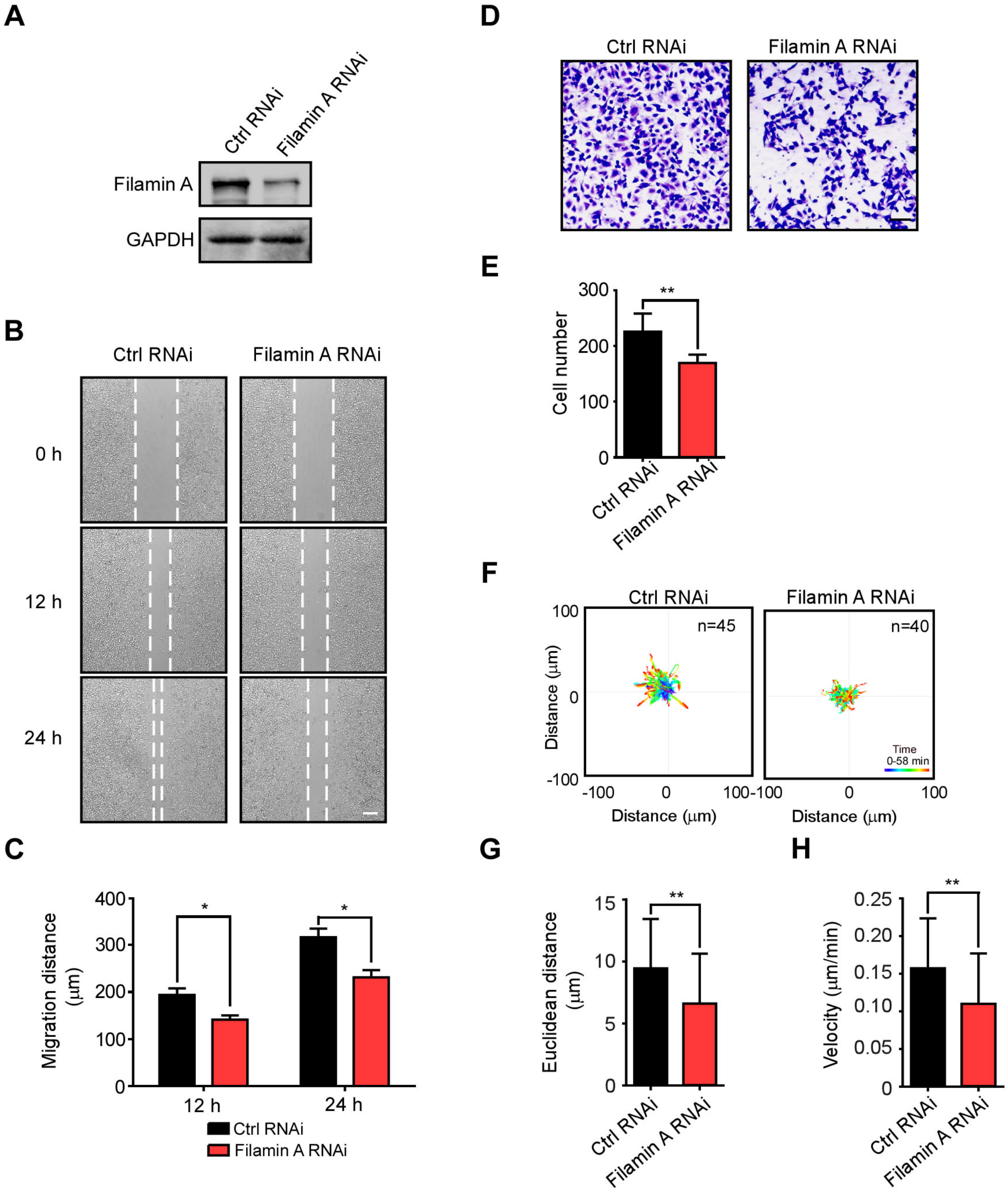

Supplementary Figure S4

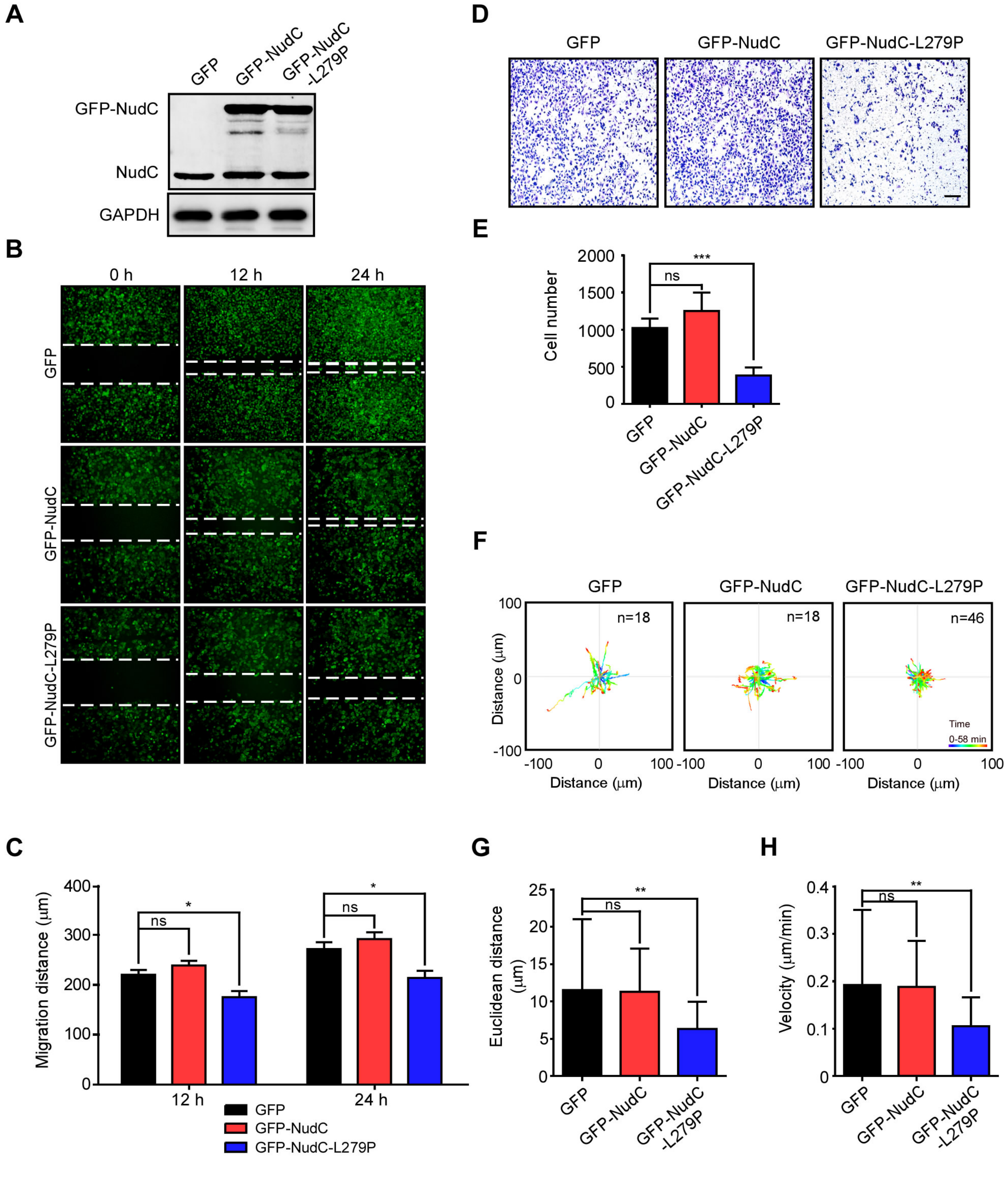

Supplementary Figure S5

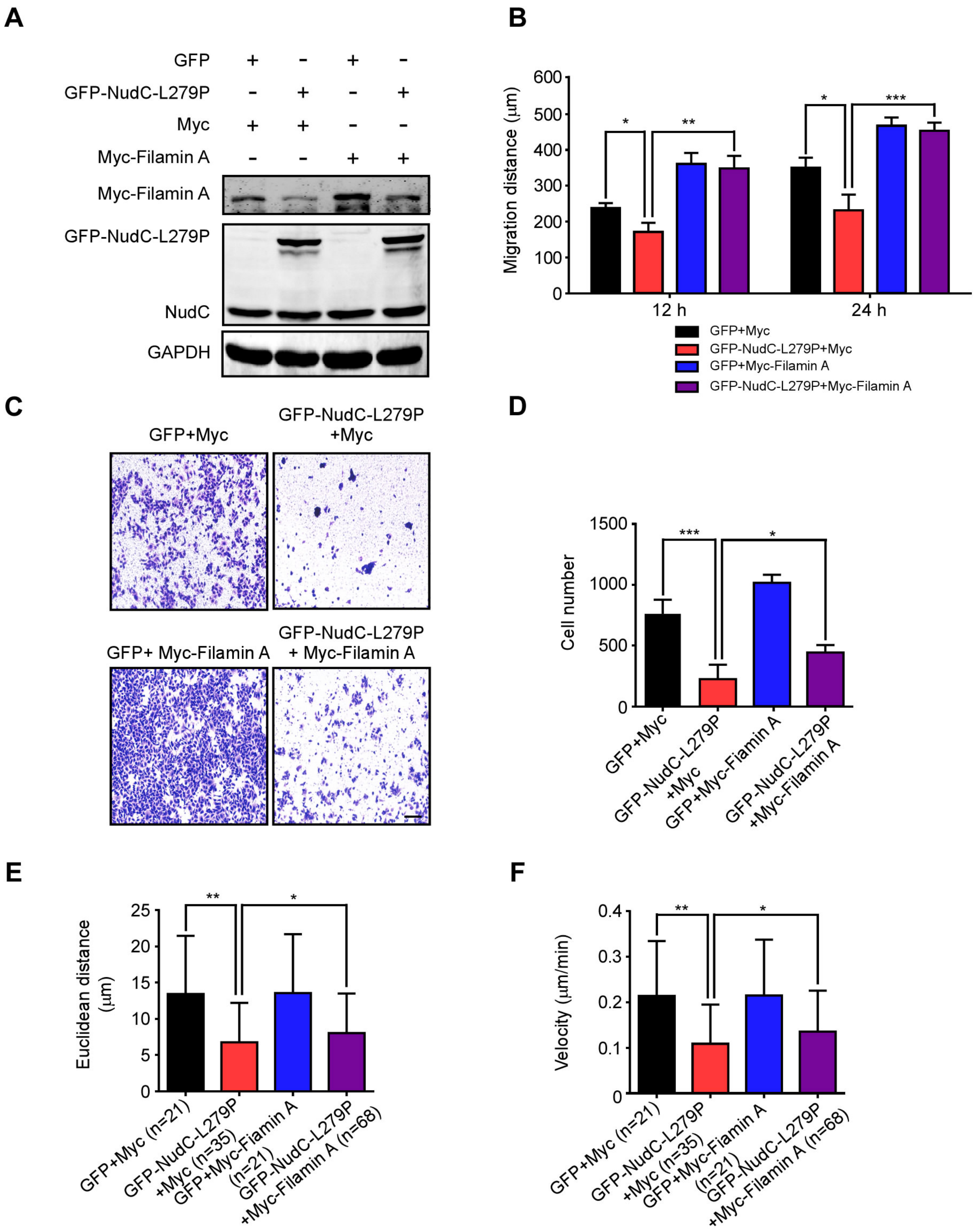

Supplementary Figure S6

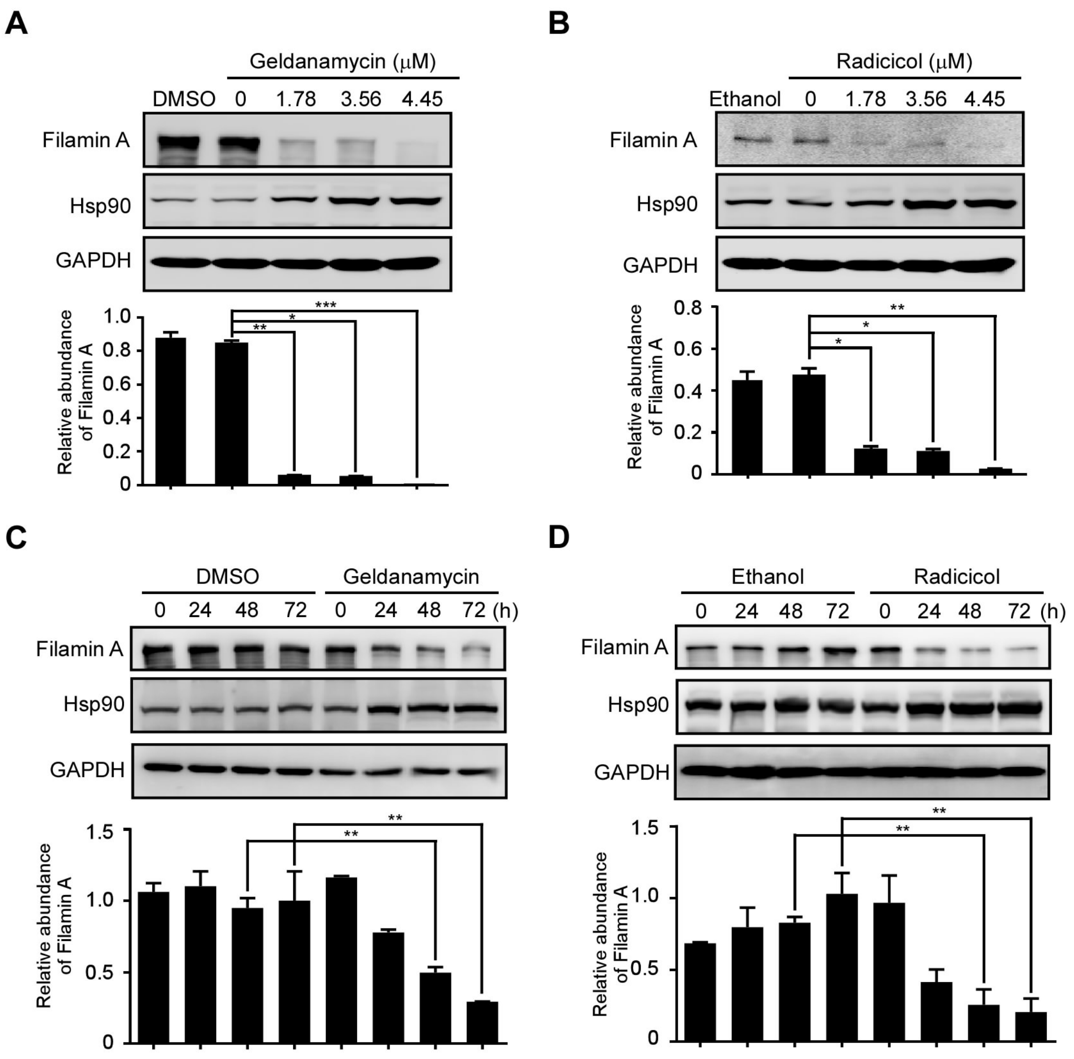

Supplementary Figure S7

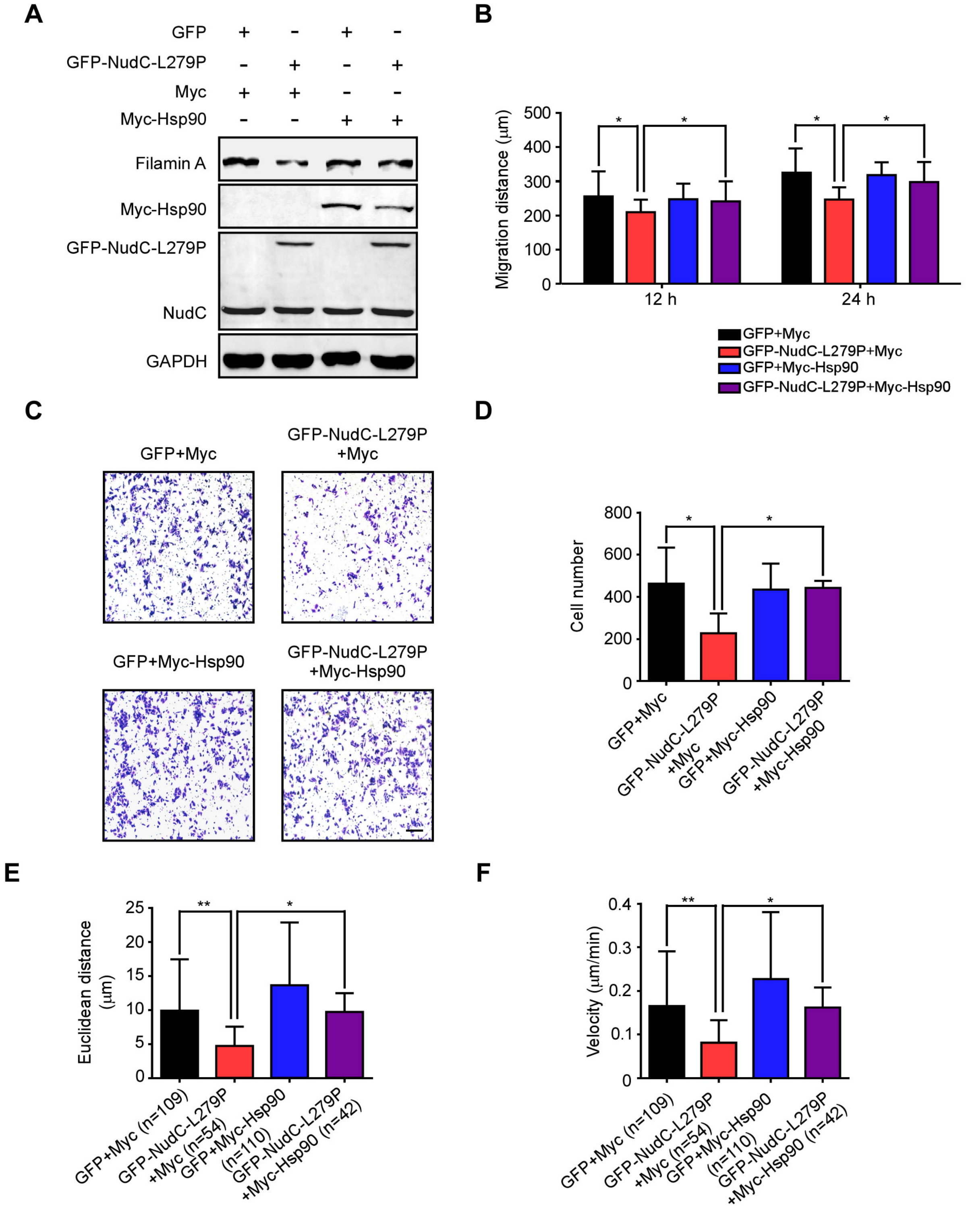

Supplementary Figure S8

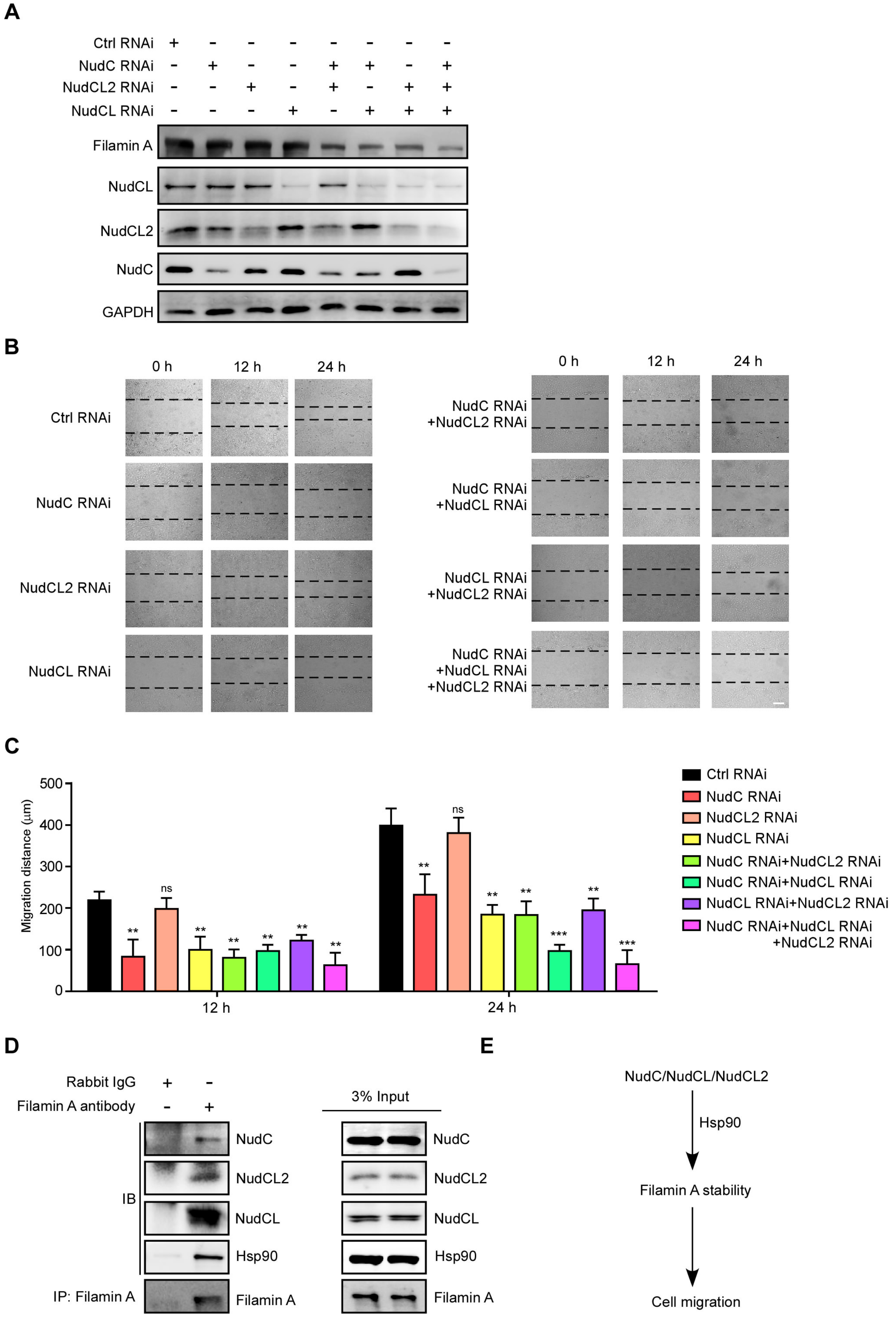

Supplementary Figure S9

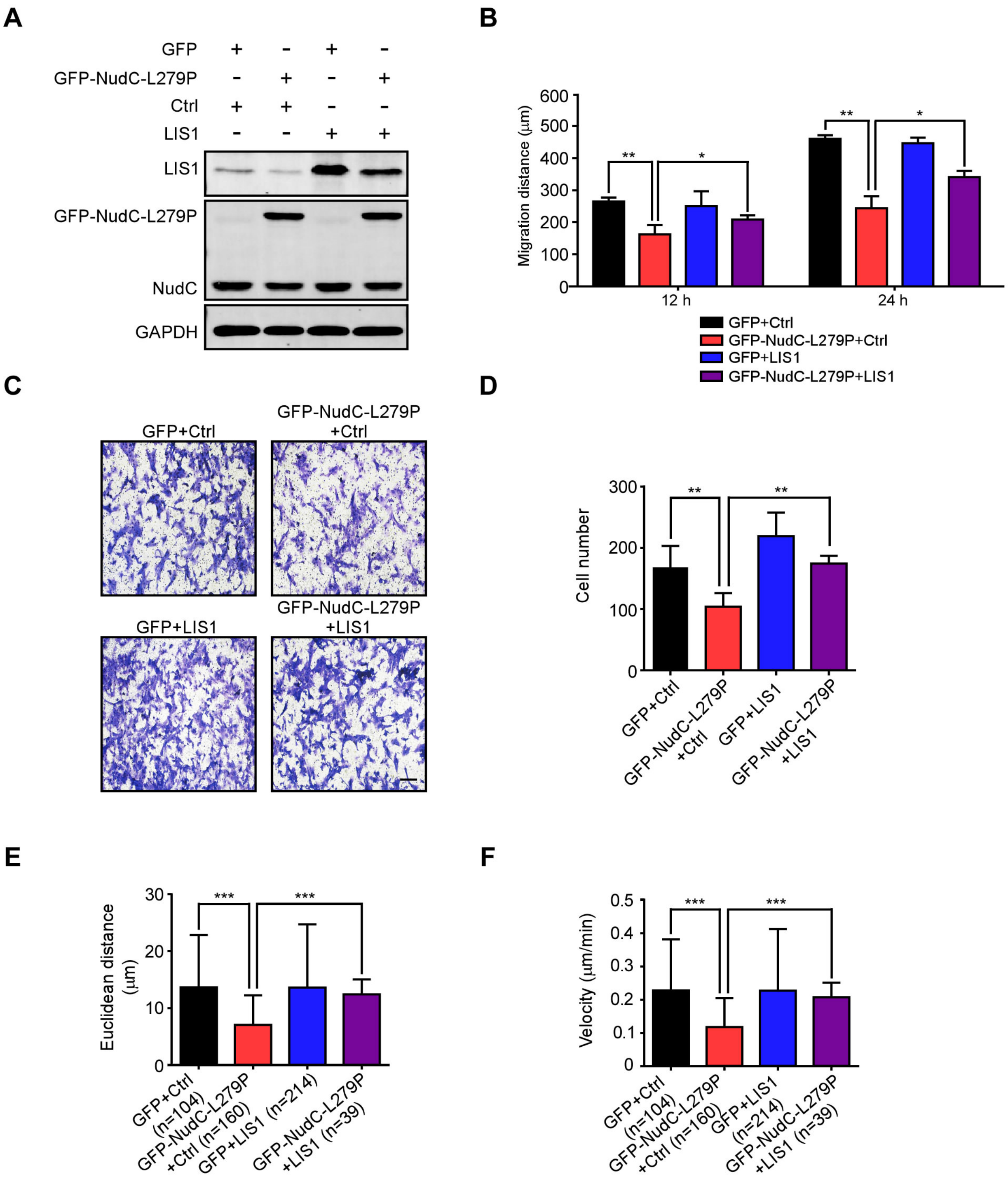

Supplementary Figure S10

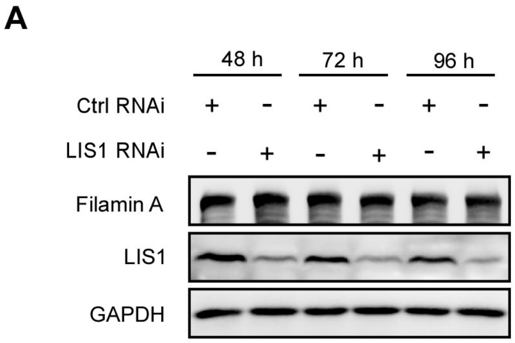

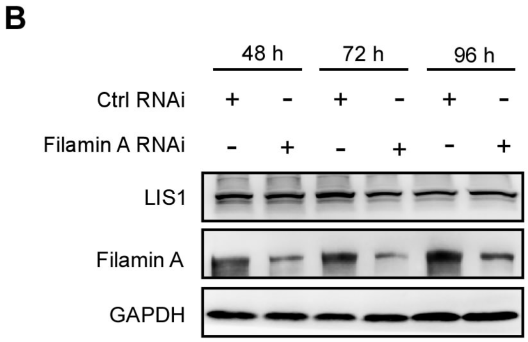

Supplement: Supplementary file 1 [file Image1.pdf]
